# Depletion of Short-Chain Fatty Acid-Producing Commensals in Gastric Cancer Is Not Detectably Modified by Type 2 Diabetes or Tumor Characteristics

**DOI:** 10.3390/metabo16100738

**Published:** 2026-09-30

**Authors:** Da-Hye Gu, Amara Zulfiqar, Sang-Ho Jeong, Hyun-Hi Kang, Hyun-Young Joo, Jung-Eun Yim

**Affiliations:** 1 Department of Food and Nutrition, Changwon National University, Changwon 51140, Republic of Korea; koodh4358@changwon.ac.kr; 2 Interdisciplinary Program in Senior Human Ecology, Changwon National University, Changwon 51140, Republic of Korea; 20247906@gs.cwnu.ac.kr; 3 Department of Surgery, Gyeongsang National University School of Medicine and Gyeongsang National University Changwon Hospital, Changwon 51472, Republic of Korea; jshgnu@gmail.com; 4 Department of Nutritional Services, Gyeongsang National University Changwon Hospital, Changwon 51472, Republic of Korea; ghqlsdl@gnuh.co.kr (H.-H.K.);

**Keywords:** gastric cancer, type 2 diabetes, gut microbiota, fecal dysbiosis, short-chain fatty acids, butyrate-producing bacteria

## Abstract

**Background/Objectives**: Gastric cancer (GC) is associated with depletion of short-chain fatty acid (SCFA)-producing gut commensals, and the same taxa are consistently reduced in type 2 diabetes (T2D). Co-existing metabolic disease might therefore be expected to compound GC-associated dysbiosis, but this has not been examined within a single cohort alongside tumor characteristics. **Methods**: Fecal microbiota of 97 Korean adults (63 with GC, 34 controls) were profiled by 16S rRNA gene sequencing. Composition, α-diversity, and β-diversity were compared between groups, and within the GC cohort by T2D status, *H. pylori* status, tumor stage, and lesion location. The summed relative abundance of significantly depleted SCFA-producing genera was analyzed as an aggregate measure of SCFA-producer abundance. Comparisons used Mann–Whitney U tests with Benjamini–Hochberg correction; community-level differences were tested by PERMANOVA. **Results**: SCFA-producer abundance fell from 26.7 ± 16.9% of the fecal community in controls to 6.6 ± 8.3% in patients with GC (*p* < 0.001; Cliff’s δ = +0.76, 95% CI +0.61 to +0.89). Enriched genera were predominantly oral and opportunistic, accompanied by expansion of *Proteobacteria*. Community-level separation was confirmed by PERMANOVA (*R*^2^ = 0.088, *p* = 0.001) with homogeneous dispersions. Within the GC cohort, SCFA-producer abundance did not differ by T2D status, *H. pylori* status, tumor stage, or lesion location (*p* = 0.125–0.332), though confidence intervals were wide. **Conclusions**: Depletion of SCFA-producing commensals in gastric cancer extends across the guild rather than individual genera and was not detectably modified by co-existing T2D or tumor characteristics. Within the limits of this cohort, the signature therefore appears to reflect the presence of disease rather than its clinical or metabolic heterogeneity.

## 1. Introduction

Gastric cancer (GC) is the fifth most commonly diagnosed malignancy and a leading cause of cancer-related death worldwide, with incidence concentrated disproportionately in East Asia; South Korea, in particular, records among the highest national incidence rates globally [1], although age-standardized incidence has declined steadily since 2011 [2]. *Helicobacter pylori* infection is the principal etiological driver of gastric carcinogenesis and is classified as a Group 1 carcinogen, initiating the stepwise Correa cascade from chronic gastritis through atrophy, intestinal metaplasia, and dysplasia to adenocarcinoma. Yet only a minority of infected individuals ultimately develop GC, a substantial proportion of cases arise in *H. pylori*-negative patients, and incidence differs markedly among populations with comparable infection prevalence [3]. These observations indicate that factors beyond *H. pylori*, including the wider gastrointestinal microbiota and the host metabolic environment, contribute meaningfully to gastric carcinogenesis and may help explain its epidemiological heterogeneity.

The gut microbiota maintains host homeostasis by fermenting dietary substrates into short-chain fatty acids (SCFAs), reinforcing epithelial barrier integrity, and regulating mucosal and systemic immunity; its disruption (dysbiosis) is increasingly implicated in gastrointestinal malignancy [4]. Across fecal microbiome studies of GC, a recurring signature has emerged: depletion of SCFA-producing commensals alongside enrichment of oral and opportunistic taxa, most consistently *Streptococcus* [4,5]. This enrichment may not be incidental. *Streptococcus anginosus* colonizes the murine stomach and induces chronic gastritis, parietal cell atrophy, metaplasia, and dysplasia, establishing at least one enriched organism as capable of driving gastric carcinogenesis rather than merely accompanying it [6]. Because butyrate produced by these commensals suppresses mucosal inflammation, supports epithelial barrier integrity [7], and can directly oppose the proliferative metabolism of gastric tumor cells by inhibiting the Warburg effect and promoting their apoptosis [4], the loss of butyrate-producing taxa represents a biologically coherent feature of the GC-associated microbial environment rather than an incidental finding.

A largely parallel body of evidence establishes these same butyrate-producing commensals as central to host metabolic health, positioning them as a candidate point of convergence between GC and metabolic disease [8,9]. Among gut bacteria, *Faecalibacterium prausnitzii*, the principal butyrate producer of the human colon, is one of the most consistently depleted taxa in type 2 diabetes (T2D), a pattern reproduced across geographically diverse cohorts and now regarded as a hallmark of the diabetic gut microbiome [10,11]. This depletion is not merely correlative: in diabetic animal models, supplementation with *F. prausnitzii* improves glucose homeostasis, ameliorates insulin resistance and hepatic steatosis, and restores intestinal barrier structure, indicating a direct contribution to metabolic regulation [12]. Mechanistically, depletion of butyrate-producing taxa compromises epithelial barrier integrity and promotes translocation of bacterial lipopolysaccharide into the circulation, a state of metabolic endotoxemia that drives the chronic low-grade inflammation and impaired insulin signaling characteristic of T2D [13,14]. Conversely, restoring butyrate availability enhances barrier function, stimulates enteroendocrine secretion of incretins such as glucagon-like peptide-1, and improves systemic insulin sensitivity [15,16]. SCFA-producing commensals thus occupy the mechanistic intersection of mucosal inflammation, epithelial integrity, and systemic glucose metabolism, the same functional domain implicated in gastric carcinogenesis.

Most microbiological work in gastric cancer has examined the gastric mucosa, which requires endoscopy and biopsy. The fecal compartment is accessible without intervention and reflects the distal consequences of a proximal disease process, but the two do not behave identically: mucosal diversity contracts across the carcinogenic sequence, whereas pooled analysis of nine fecal cohorts found compositional change without a consistent diversity difference [17]. The connection between them is direct, as oral and upper gastrointestinal organisms reach and colonize the distal gut, particularly where the resident community is already perturbed [18].

This shared dependence on butyrate-producing commensals raises the possibility that gastric cancer and T2D converge on a common microbial axis, such that their co-existence in the same patient could further compound the depletion observed in gastric cancer. The clinical plausibility of such an interaction is supported by epidemiological evidence. Meta-analyses report a modest increase in GC risk among individuals with T2D (relative risk ≈ 1.19) that persists in East Asian populations and after adjustment for *H. pylori* infection and smoking [19]; individual-participant pooled data localize this excess risk to the cardia [20], the subsite most closely tied to obesity and systemic metabolic dysfunction; and a territory-wide Asian cohort has shown that T2D raises GC risk even after *H. pylori* eradication [21], implicating a metabolic contribution independent of infection. Despite this mechanistic and epidemiological convergence, GC-associated and T2D-associated dysbiosis have almost always been characterized in isolation: gastric microbiome studies have rarely stratified patients by metabolic comorbidity, and T2D microbiome studies have not addressed concurrent malignancy. Consequently, whether co-existing T2D independently reshapes the GC-associated fecal microbiome, beyond the alterations attributable to cancer alone, remains unclear, as does how this potential metabolic modifier compares with established GC-associated variables such as *H. pylori* status, tumor stage, and lesion location.

To address this gap, we characterized the fecal microbiota of Korean adults with gastric cancer and healthy controls by 16S rRNA gene amplicon sequencing of the V3–V4 region, comparing taxonomic composition, alpha diversity, and community structure between groups and, within the GC cohort, across T2D status, *H. pylori* status, tumor stage, and primary lesion location. Depleted SCFA-producing genera were additionally analyzed as an aggregate measure, allowing the fermentative guild to be assessed as a functional unit rather than as individual taxa. We hypothesized that co-existing T2D would further perturb GC-associated dysbiosis, particularly among SCFA-producing commensals linked to metabolic homeostasis, and that systemic metabolic status would shape the fecal microbiome more closely than local tumor characteristics.

## 2. Materials and Methods

### 2.1. Study Design and Participants

This cross-sectional study enrolled 97 adults (aged ≥19 years), comprising 34 healthy controls and 63 patients with histologically confirmed gastric cancer (GC). Recruitment took place between May 2018 and March 2021. Healthy controls were enrolled from Changwon National University and Changwon Fatima Hospital, and patients with confirmed gastric cancer from Gyeongsang National University Changwon Hospital. Eligibility for the control group required the absence of gastrointestinal disease, malignancy, type 2 diabetes, and chronic use of any medication known to alter the gut microbiota. Control participants were additionally restricted to those aged ≥45 years to reduce the age disparity between groups. Participants who had received antibiotics, probiotics, or acid-suppressive therapy within the four weeks preceding sample collection were excluded. Patients with GC were included based on histologically confirmed diagnosis and were subsequently stratified as non-diabetic (*n* = 46) or diabetic (*n* = 17) according to standard diagnostic criteria. Patients who had undergone chemotherapy or biological therapy before providing a stool sample, or who reported recent antibiotic or probiotic use within the same four-week window, were also excluded.

### 2.2. Anthropometric and Biochemical Measurements

Anthropometric measurements were performed by a trained examiner with participants wearing light clothing and no shoes. Body mass index (BMI) was calculated as body weight (kg) divided by height squared (m^2^). Following an overnight fast of at least 12 h, a single venous blood sample was drawn from each participant and used for both routine biochemistry and *H. pylori* serology. Serum was separated by centrifugation at 1500× *g* for 15 min and stored at −80 °C until analysis. Fasting plasma glucose was measured by an enzymatic method using a commercial kit (Asan Pharmaceutical, Seoul, Republic of Korea). Glycated hemoglobin (HbA1c) was determined enzymatically using a commercial assay (Crystal Chem, Downers Grove, IL, USA). Total cholesterol, aspartate aminotransferase (AST), and alanine aminotransferase (ALT) were quantified on an automated clinical chemistry analyzer at the hospital’s central laboratory according to standard clinical procedures.

### 2.3. Diabetes Diagnostic Criteria

Type 2 diabetes was diagnosed in accordance with the American Diabetes Association (ADA) Standards of Medical Care in Diabetes [22], requiring fulfilment of at least one of the following criteria: glycated hemoglobin (HbA1c) ≥ 6.5%; fasting plasma glucose ≥ 126 mg/dL following ≥8 h of fasting; 2-h plasma glucose ≥ 200 mg/dL during a 75 g oral glucose tolerance test; or a random plasma glucose ≥ 200 mg/dL in the presence of classical hyperglycemic symptoms (polyuria, polydipsia, or unintended weight loss).

### 2.4. Clinical Classification of Patients with Gastric Cancer

Patients with gastric cancer were further classified according to *H. pylori* infection status, primary tumor lesion location, and tumor stage. Because complete data for these variables were not uniformly available across the GC cohort, sub-analyses were restricted to patients with the requisite information for each classification (*H. pylori*, *n* = 48; tumor stage, *n* = 62; lesion location, *n* = 59), with the corresponding sample sizes indicated in Section 3.

*H. pylori* infection status. Anti-*Helicobacter pylori* IgG antibodies were measured in the serum sample by enzyme-linked immunosorbent assay (ELISA), with confirmation by immunochromatographic testing where indicated. Patients were classified as *H. pylori*-positive or *H. pylori*-negative according to the manufacturer’s standard cut-off values.

Tumor lesion location. Primary tumor lesion location was determined by upper gastrointestinal endoscopy at the time of diagnosis, with malignancy confirmed by histopathological examination of endoscopic biopsy specimens. Lesions were categorized by gastric region as upper (cardia and fundus), middle (body), or lower (antrum and pylorus).

Tumor stage. Tumor stage was determined by computed tomography (CT) at the time of diagnosis, complemented by endoscopic ultrasonography, magnetic resonance imaging (MRI), and positron emission tomography (PET) where clinically indicated. Tumors were classified according to the Union for International Cancer Control/American Joint Committee on Cancer (UICC/AJCC) TNM staging system, 7th edition, into Stages I through IV. For stratified analysis, patients were dichotomized into Stage I and Stage ≥ II groups.

### 2.5. Stool Sample Collection and DNA Extraction

Fresh fecal samples were collected on-site using sterile containers and processed immediately following collection. Each sample was aseptically aliquoted and flash-frozen at −80 °C to preserve microbial community integrity, and subsequently transported on dry ice to AiBiotics Co., Ltd. (Seoul, Republic of Korea) for microbiome analysis. Approximately 50–100 mg of each specimen was homogenized by mechanical bead-beating to ensure efficient microbial cell lysis, and genomic DNA was extracted using the FastDNA™ SPIN Kit for Soil (MP Biomedicals, Irvine, CA, USA) in accordance with the manufacturer’s protocol. DNA yield and purity were assessed spectrophotometrically prior to downstream processing. Identical collection, storage, and extraction procedures were applied across all participants to minimize technical variability.

### 2.6. 16S rRNA Gene Amplification and Sequencing

The V3–V4 region of the bacterial 16S rRNA gene was amplified using the primers 341F (5′-CCTACGGGNGGCWGCAG-3′) and 805R (5′-GACTACHVGGGTATCTAATCC-3′) [23], each carrying Illumina overhang adapter sequences. Polymerase chain reaction (PCR) was performed in 25 µL reactions containing 2× KAPA HiFi HotStart ReadyMix (Kapa Biosystems, Wilmington, MA, USA), 0.2 µM of each primer, and approximately 10 ng of template DNA. Thermal cycling consisted of initial denaturation at 95 °C for 3 min, followed by 25 cycles of denaturation at 95 °C for 30 s, annealing at 55 °C for 30 s, and extension at 72 °C for 30 s, with a final extension at 72 °C for 5 min. Amplification was confirmed by 1% agarose gel electrophoresis visualized on a Gel Doc™ system (Bio-Rad, Hercules, CA, USA). PCR products were purified with CleanPCR magnetic beads (CleanNA, Waddinxveen, The Netherlands), pooled at equimolar concentrations, and assessed for library integrity on a Bioanalyzer 2100 (Agilent Technologies, Palo Alto, CA, USA). Extraction and no-template PCR controls were processed in parallel and confirmed the absence of contaminating amplification. Sequencing was performed on the Illumina MiSeq platform using the MiSeq Reagent Kit v3 (2 × 300 bp paired-end; Illumina, Inc., San Diego, CA, USA) according to the manufacturer’s protocol, with a 20% PhiX control spike-in included in the pooled library to enhance base diversity during sequencing.

### 2.7. Bioinformatic Analysis

Raw paired-end sequence reads were processed by AiBiotics Co., Ltd. (Seoul, Republic of Korea) using standard 16S rRNA analytical workflows. Low-quality reads, chimeric sequences, and reads shorter than 300 bp were removed. High-quality reads were clustered into operational taxonomic units (OTUs) at a 97% sequence-similarity threshold using UCLUST, and taxonomic classification was assigned against the EzBioCloud 16S rRNA gene database (ChunLab Inc., Seoul, Republic of Korea). Samples with sequencing depths below 10,000 reads were excluded and read counts were rarefied to the minimum sample depth to normalize sequencing coverage across samples. Sufficient depth for genus-level community characterization was achieved across all retained samples. Low-abundance OTUs accounting for less than 0.005% of total read counts were removed prior to downstream analysis. Alpha diversity indices, including observed OTUs, Chao1 richness, and the Shannon diversity index, were computed from the rarefied OTU table and additionally summarized at the phylum and genus levels by collapsing OTU counts to the corresponding taxonomic ranks. Between-group differences in alpha diversity were evaluated using independent two-sample *t*-tests. Beta diversity was assessed using Bray–Curtis dissimilarity and visualized through principal coordinates analysis (PCoA) and non-metric multidimensional scaling (NMDS).

### 2.8. Statistical Analysis

All statistical analyses were performed using SPSS Statistics version 27.0 (IBM Corp., Armonk, NY, USA) and R version 4.0.3 (R Foundation for Statistical Computing, Vienna, Austria). Continuous variables are presented as mean ± standard deviation (SD), and categorical variables as frequencies and percentages. The distributional normality of continuous variables was assessed using the Shapiro–Wilk test, and homogeneity of variance was evaluated using Levene’s test. Between-group comparisons of continuous variables were performed using the independent two-sample *t*-test for normally distributed data, with Welch’s correction applied in cases of unequal variance, and categorical variables were compared using the chi-square (χ^2^) test. For variables that deviated substantially from normality, Mann–Whitney U tests were used as a non-parametric alternative. For comparisons across three groups (primary tumor lesion location), one-way analysis of variance (ANOVA) was used. Relative abundances of bacterial taxa at the phylum and genus levels were compared using the Mann–Whitney U test, or the Kruskal–Wallis test for three-group comparisons. SCFA-producer abundance was defined as the summed relative abundance of eight genera depleted in gastric cancer and recognized as short-chain fatty acid producers or as acetate- and lactate-supplying cross-feeders within the colonic fermentation network: *Faecalibacterium*, *Bifidobacterium*, *Dorea*, *Subdoligranulum*, *Blautia*, *Anaerostipes*, *Fusicatenibacter*, and *Lachnospira*. This aggregate was derived from the taxa identified in the present cohort and was not prespecified as a diagnostic biomarker panel. For all analyses, a two-sided *p*-value of <0.05 was considered statistically significant. Effect sizes for comparisons of microbial abundance are reported as Cliff’s delta for two-group comparisons and epsilon-squared (ε^2^) for three-group comparisons, each with bootstrap 95% confidence intervals (2000 resamples). For between-group comparisons of relative bacterial abundance across multiple taxa, Benjamini–Hochberg false discovery rate (FDR) correction was applied within each family of tests, with phylum- and genus-level comparisons corrected separately; FDR-adjusted q-values are provided in Supplementary Tables S1 and S2. Rank-based tests on relative abundances do not formally account for the compositional structure of microbiome data and may inflate false discovery rates [24]. All between-group comparisons of microbial abundance were therefore repeated on centered log-ratio (CLR)-transformed abundances, restricted to taxa present in at least 10% of samples, with zero values replaced by a multiplicative correction. Community-level differences in microbial composition were tested by permutational multivariate analysis of variance (PERMANOVA) on Bray–Curtis dissimilarity matrices with 999 permutations, and homogeneity of multivariate dispersion was assessed by PERMDISP. Sensitivity analyses were performed in an age-matched subset and by comparing age tertiles within the GC cohort. Associations of age and of metabolic variables with SCFA-producer abundance were assessed by Spearman correlation within each group.

## 3. Results

### 3.1. Baseline Characteristics of the Study Cohort

A total of 97 adults were enrolled, comprising 34 healthy controls and 63 patients with histologically confirmed gastric cancer (GC). Within the GC cohort, 46 patients were classified as non-diabetic and 17 as diabetic. Sex distribution did not differ significantly between controls and patients with GC (*p* = 0.184). Patients with GC were significantly older than controls (62.54 ± 12.59 vs. 50.85 ± 3.80 years; *p* < 0.001), whereas body weight and body mass index (BMI) were comparable between the two groups (*p* = 0.675 and *p* = 0.996, respectively). Among biochemical markers, fasting glucose, total cholesterol, and aspartate aminotransferase (AST) levels did not differ significantly between groups, whereas alanine aminotransferase (ALT) was significantly higher in patients with GC than in controls (25.52 ± 18.04 vs. 10.47 ± 7.43 IU/L; *p* < 0.001). Detailed demographic and biochemical characteristics are presented in Table 1.

### 3.2. Gastric Cancer Is Associated with Guild-Level Depletion of SCFA-Producing Commensals

At the phylum level, *Firmicutes* (*p* = 0.006) and *Actinobacteria* (*p* = 0.048) were less abundant in patients with GC than in healthy controls, while *Proteobacteria*, *Fusobacteria*, *Verrucomicrobia*, and *Synergistetes* were enriched (all *p* < 0.001). The largest shift was in *Proteobacteria*, which rose from 1.84 ± 2.97% in controls to 12.94 ± 15.87% in patients (Cliff’s δ = −0.63, 95% CI −0.79 to −0.44). The *Firmicutes*-to-*Bacteroidetes* ratio did not differ significantly between groups (*p* = 0.080), and the remaining phyla were comparable (Supplementary Table S1).

Eight short-chain fatty acid (SCFA)-producing genera were significantly depleted in the GC group: *Subdoligranulum*, *Blautia*, *Anaerostipes*, *Faecalibacterium*, *Bifidobacterium*, *Fusicatenibacter*, and *Lachnospira* (all *p* < 0.001), and *Dorea* (*p* = 0.003). In contrast, *Streptococcus*, *Eubacterium*, *Klebsiella*, *Veillonella*, *Enterobacter*, and *Fusobacterium* (all *p* < 0.001) and *Prevotella* (*p* = 0.012) were enriched in patients with GC. All fifteen differences survived Benjamini–Hochberg FDR correction and fourteen of fifteen were reproduced on centered log-ratio-transformed abundances (Supplementary Table S1). Considered as a group, SCFA-producing genera accounted for 26.71 ± 16.92% of the fecal community in controls and 6.55 ± 8.25% in patients with GC (*p* < 0.001; Cliff’s δ = +0.76, 95% CI +0.61 to +0.89), indicating depletion across the guild rather than loss of a single genus (Table 2).

**Figure 1:**
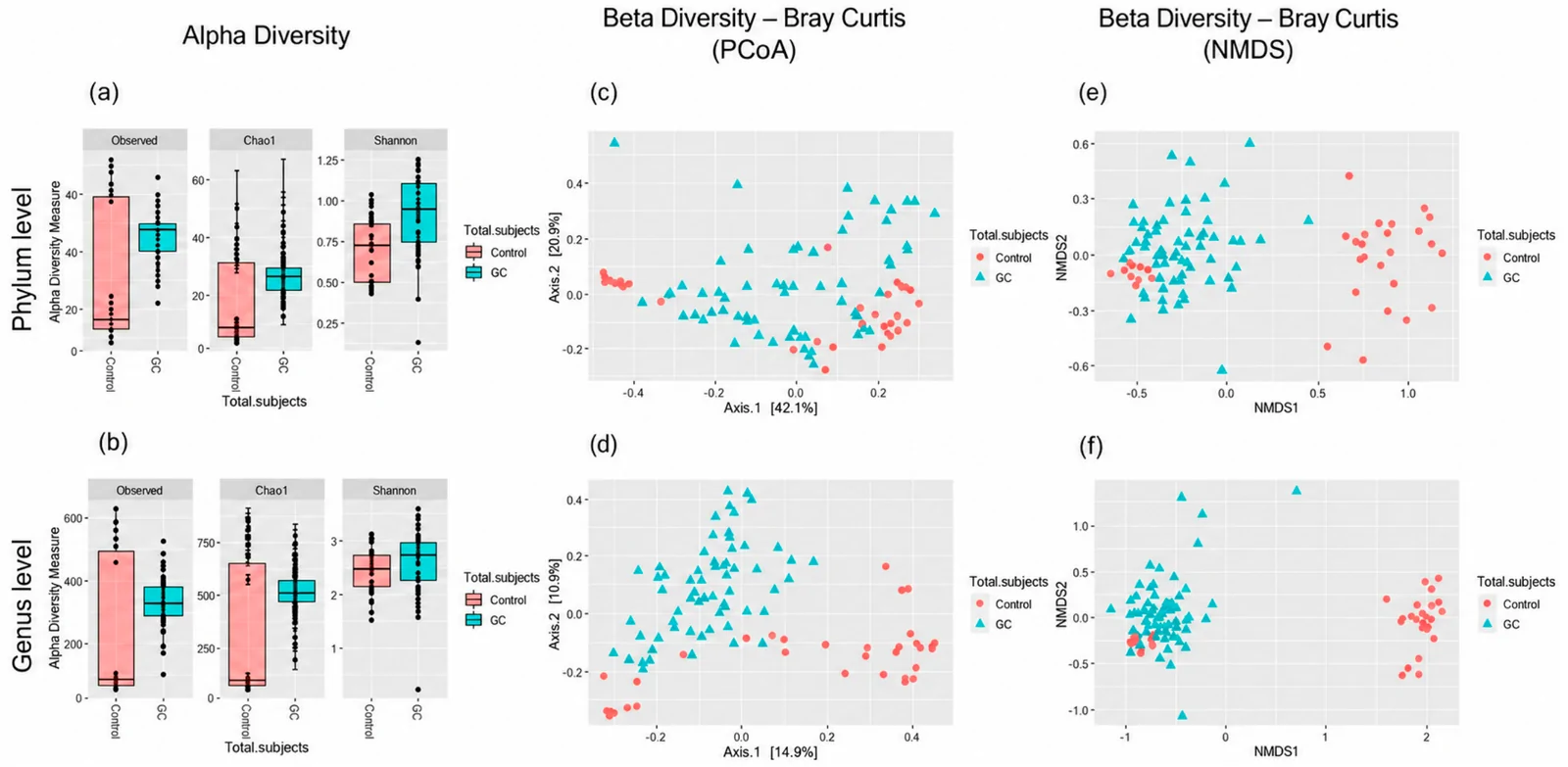
Comparison of gut microbial diversity between healthy controls and patients with gastric cancer at the phylum (top row) and genus (bottom row) levels. Alpha diversity indices (observed OTUs, Chao1 richness, Shannon diversity) at the phylum (**a**) and genus (**b**) levels. Beta diversity visualized by Bray–Curtis PCoA at the phylum (**c**) and genus (**d**) levels. Bray–Curtis NMDS ordination at the phylum (**e**) and genus (**f**) levels.

Alpha diversity analysis revealed greater microbial richness in patients with GC than in controls. At the phylum level, observed OTUs, the Chao1 index, and the Shannon index were all significantly elevated in the GC group (*p* = 0.001, *p* < 0.001, and *p* < 0.001, respectively). At the genus-level, the observed OTUs and Chao1 index remained higher in patients with GC (*p* = 0.005 and *p* < 0.001, respectively), whereas the Shannon index did not differ significantly between groups (*p* = 0.139), indicating that increased taxonomic richness in GC was not accompanied by a proportional increase in community evenness (Figure 1). Bray–Curtis-based principal coordinates analysis (PCoA) and non-metric multidimensional scaling (NMDS) revealed visual separation between the two groups at the genus-level (Figure 1), supporting overall compositional differences in gut microbial community structure between patients with GC and healthy controls. This separation was confirmed statistically by PERMANOVA (pseudo-*F* = 9.20, *R*^2^ = 0.088, *p* = 0.001), with homogeneous group dispersions (PERMDISP *p* = 0.297), indicating a genuine compositional difference rather than unequal within-group variability.

SCFA-producer abundance was unrelated to age within controls (rho = −0.002, *p* = 0.990) but was inversely associated with age within the GC cohort (rho = −0.307, *p* = 0.014). Analyses were therefore repeated in an age-matched subset spanning 45–58 years (34 controls, 17 patients with GC; *p* = 0.495 for age), in which the difference in SCFA-producer abundance persisted (26.71% vs. 8.55%; *p* = 1.5 × 10^−4^, Cliff’s δ = +0.66). Within the GC cohort, age tertiles spanning 31–91 years showed no compositional differences (*R*^2^ = 0.038, *p* = 0.243).

### 3.3. SCFA-Producer Abundance in Patients with Gastric Cancer Stratified by Type 2 Diabetes

Of the 63 patients with GC, 46 were classified as non-diabetic and 17 as diabetic. Demographic and anthropometric variables, including sex distribution, age, body weight, and BMI, were comparable between the two subgroups (Table 3). Among biochemical markers, fasting glucose was higher in diabetic patients than in non-diabetic patients but did not reach statistical significance (*p* = 0.087). Total cholesterol, in contrast, was significantly lower in diabetic patients (*p* = 0.035), while AST and ALT levels showed no significant difference between the two subgroups.

None of the eight SCFA-producing genera differed significantly between diabetic and non-diabetic patients with GC. SCFA-producer abundance was lower in the diabetic subgroup (4.10 ± 3.87% vs. 7.45 ± 9.24%), but this difference did not reach statistical significance (*p* = 0.152), and the confidence interval around the effect size was wide (Cliff’s δ = +0.24, 95% CI −0.07 to +0.54), spanning values consistent with no difference and with a moderate reduction. Across all genus-level comparisons, *Enterobacter* (0.12 ± 0.20% vs. 1.20 ± 3.30%; *p* = 0.029) and *Veillonella* (1.36 ± 3.64% vs. 2.03 ± 3.23%; *p* = 0.027) were lower in diabetic patients, but neither survived Benjamini–Hochberg FDR correction (Supplementary Table S2). With 17 diabetic patients, these analyses had limited power to detect differences in the magnitude observed.

**Figure 2:**
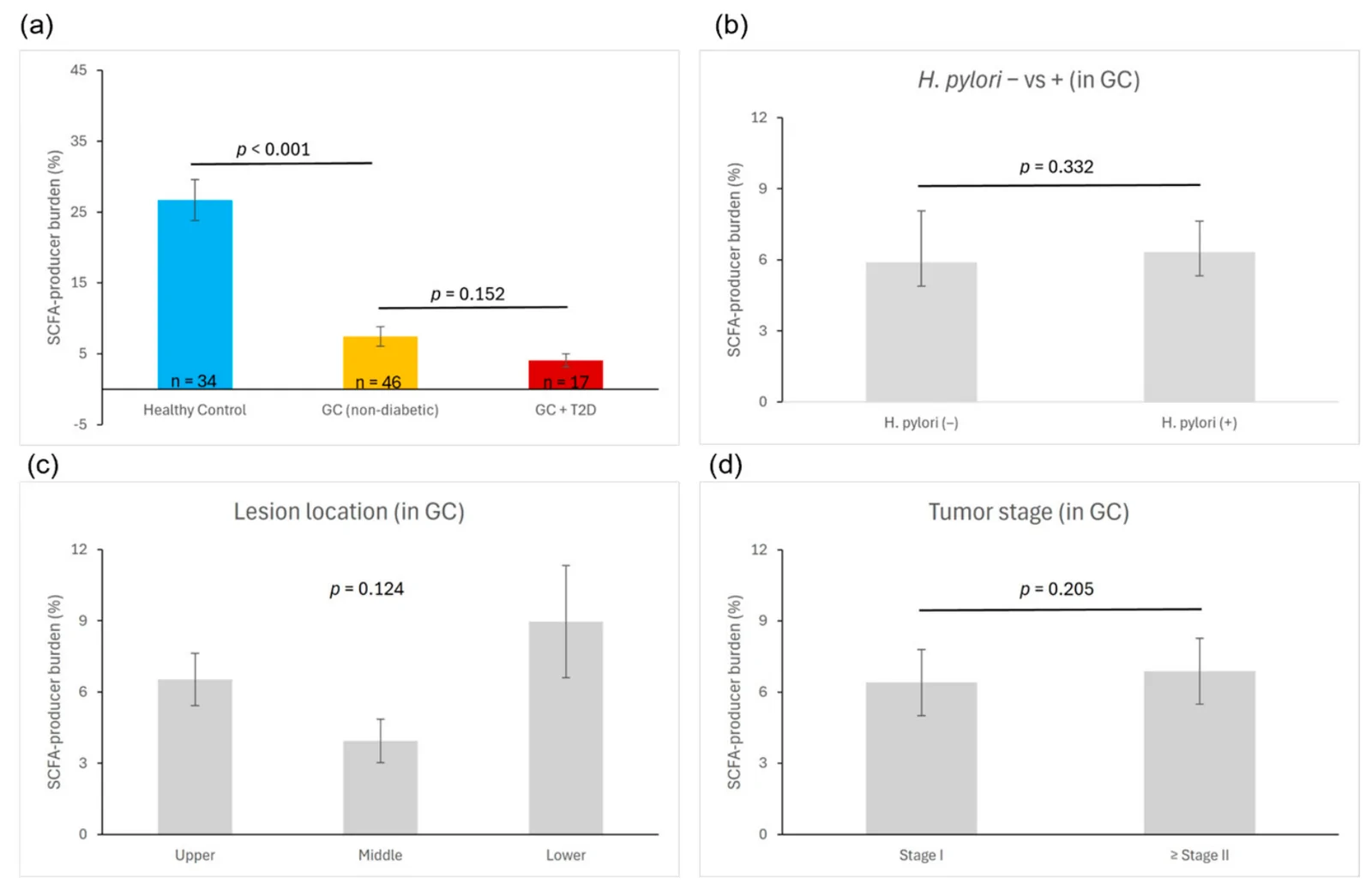
SCFA-producer abundance across clinical stratifications in gastric cancer. (**a**) Summed relative abundance of the SCFA-producer panel across healthy controls (*n* = 34), non-diabetic patients with gastric cancer (GC, *n* = 46), and patients with GC and co-existing type 2 diabetes (GC + T2D, *n* = 17); *p* < 0.001 for healthy controls versus non-diabetic GC and *p* = 0.152 for non-diabetic GC versus GC + T2D. (**b**) SCFA-producer abundance in patients with GC stratified by *Helicobacter pylori* infection status (negative, *n* = 25; positive, *n* = 23); *p* = 0.332. (**c**) SCFA-producer abundance stratified by primary tumor lesion location (upper, *n* = 16; middle, *n* = 18; lower, *n* = 25); *p* = 0.125. (**d**) SCFA-producer abundance stratified by tumor stage (Stage I, *n* = 43; ≥Stage II, *n* = 19); *p* = 0.205. Bars represent mean summed relative abundance (%) with standard error bars. *p*-values from Mann–Whitney U tests (two-group comparisons) or the Kruskal–Wallis test (three-group comparison in panel (**c**)). Group sample sizes are indicated within each bar.

When considered alongside the GC-versus-control comparison, SCFA-producer abundance declined progressively across the three groups: 26.71 ± 16.92% in healthy controls, 7.45 ± 9.24% in non-diabetic patients with GC, and 4.10 ± 3.87% in patients with GC and co-existing type 2 diabetes (Figure 2a; Kruskal–Wallis *p* < 0.001; ordered trend rho = −0.713, *p* < 0.001). The reduction from healthy controls to non-diabetic GC was significant (*p* < 0.001), whereas the further reduction in diabetic patients did not reach significance (*p* = 0.152).

**Figure 3:**
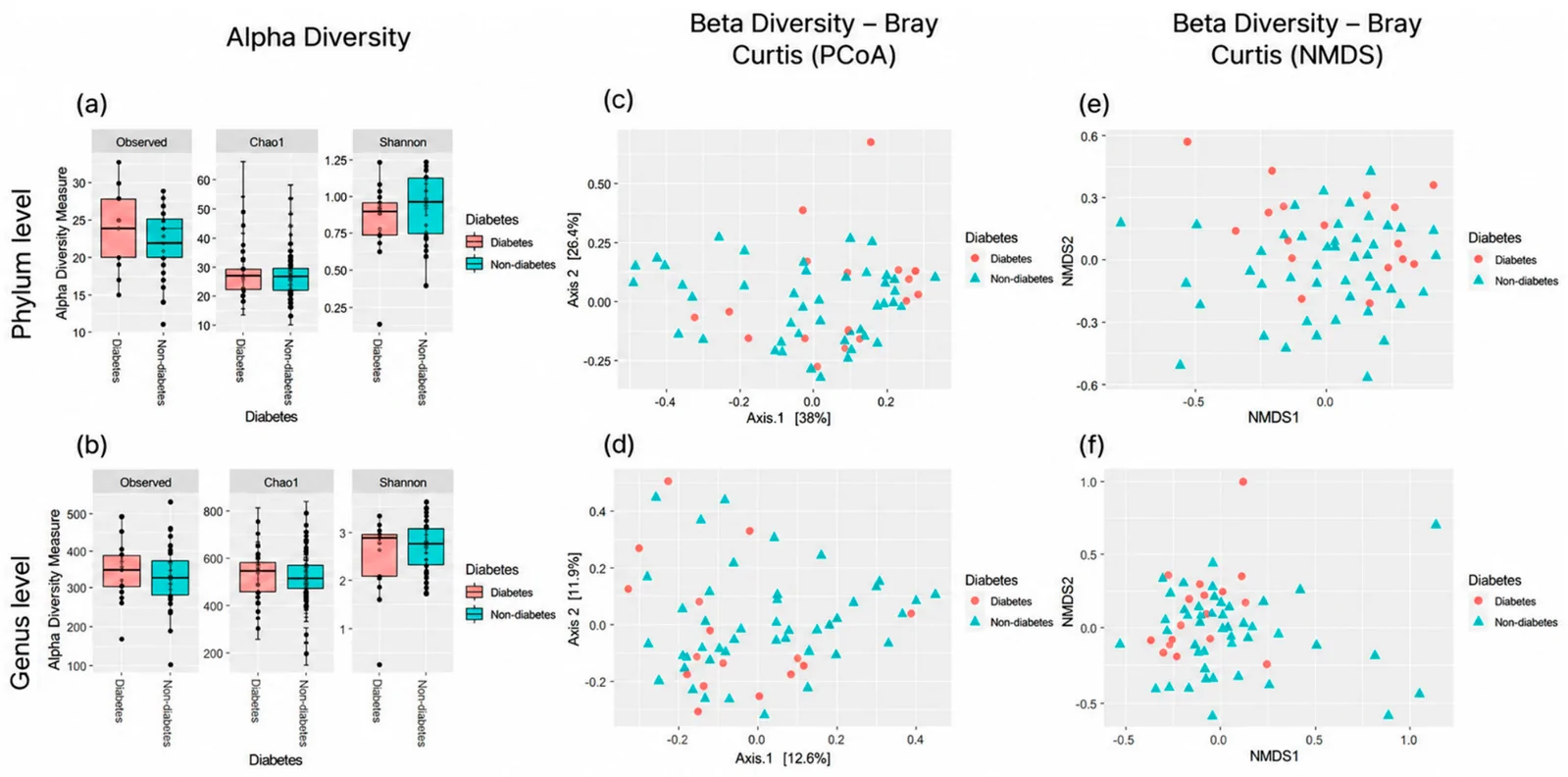
Comparison of gut microbial diversity between patients with and without diabetes at the phylum (top row) and genus (bottom row) levels. Alpha diversity indices (observed OTUs, Chao1 richness, Shannon diversity) at the phylum (**a**) and genus (**b**) levels. Beta diversity visualized by Bray–Curtis PCoA at the phylum (**c**) and genus (**d**) levels. Bray–Curtis NMDS ordination at the phylum (**e**) and genus (**f**) levels.

Alpha diversity analysis did not reveal significant differences between non-diabetic and diabetic patients with GC. At the phylum level, observed OTUs, the Chao1 index, and the Shannon index were comparable between subgroups (*p* = 0.333, *p* = 0.328, and *p* = 0.083, respectively). Genus-level alpha diversity indices were similarly unchanged (*p* = 0.579, *p* = 0.987, and *p* = 0.322 for observed OTUs, Chao1, and Shannon, respectively). Bray–Curtis-based PCoA and NMDS ordinations did not show clear group separation according to diabetes status at either taxonomic level, consistent with the absence of taxon-level differences within the GC cohort (Figure 3). PERMANOVA confirmed the absence of community-level separation by diabetes status (pseudo-*F* = 1.07, *R*^2^ = 0.017, *p* = 0.358; PERMDISP *p* = 0.429). Within controls, SCFA-producer abundance was positively associated with fasting glucose (rho = +0.401), AST (rho = +0.499), and ALT (rho = +0.422; all *p* < 0.05), whereas no association was detected within the GC cohort (Supplementary Table S3).

### 3.4. Gut Microbial Composition by Helicobacter pylori Status, Tumor Stage, and Tumor Lesion Location

To determine whether the GC dysbiosis signature was further modified by clinical sub-classifications beyond diabetes status, gut microbial composition and host clinical characteristics were examined across three additional stratifications within the GC cohort: *H. pylori* infection status, tumor stage, and primary tumor lesion location.

Stratification by *H. pylori* status (negative, *n* = 25; positive, *n* = 23) revealed no significant differences in demographic or biochemical variables, or in phylum-level relative abundances (Table 4). SCFA-producer abundance was comparable between groups (6.32 ± 6.29% vs. 5.89 ± 10.90%; *p* = 0.332; Cliff’s δ = −0.17, 95% CI −0.50 to +0.16). Among individual SCFA-producing genera, only *Faecalibacterium* differed, being more abundant in *H. pylori*-positive than in *H. pylori*-negative patients (4.06 ± 4.95% vs. 1.53 ± 1.51%; *p* = 0.039). This isolated finding did not survive Benjamini–Hochberg FDR correction and occurred against an unchanged SCFA-producer guild and is therefore regarded as exploratory (Supplementary Table S4).

Comparison of patients with Stage I (*n* = 43) and ≥Stage II (*n* = 19) disease showed that AST levels were higher in Stage I patients (30.21 ± 16.06 vs. 22.26 ± 8.35 IU/L; *p* = 0.047), whereas other anthropometric and biochemical variables did not differ between groups (Table 5). No phylum- or genus-level differences were observed, and SCFA-producer abundance was comparable between groups (6.40 ± 9.21% vs. 6.88 ± 6.03%; *p* = 0.205; Cliff’s δ = −0.20, 95% CI −0.48 to +0.09; Supplementary Table S5).

Stratification by primary tumor lesion location (upper, *n* = 16; middle, *n* = 18; lower, *n* = 25) revealed significant differences in host anthropometric characteristics (Table 6). Body weight (74.93 ± 10.78, 65.43 ± 10.75, and 61.75 ± 10.82 kg, respectively; *p* = 0.001), BMI (26.44 ± 3.81, 24.06 ± 2.84, and 23.72 ± 2.42 kg/m^2^; *p* = 0.016), and male predominance (81.25%, 77.78%, and 48.00%; *p* = 0.041) were all higher in patients with upper-lesion tumors compared with middle- and lower-lesion tumors. Despite these substantial differences in host phenotype, no significant differences in phylum- or genus-level microbial composition were observed across the three lesion locations, and SCFA-producer abundance did not differ (6.53 ± 4.41%, 3.94 ± 3.87%, and 8.97 ± 11.81%; *p* = 0.125; Supplementary Table S6). Neither alpha diversity indices nor beta diversity ordinations differed significantly across *H. pylori* status, tumor stage, or lesion location stratifications (Supplementary Figures S1, S2 and S3, respectively). PERMANOVA confirmed the absence of community-level separation for all three, with homogeneous dispersions in each case; results for all five comparisons are summarized in Table 7. No taxon differed significantly by any stratification under compositional analysis (all *q* > 0.13; Supplementary Tables S2 and S4–S6).

Considered together with the T2D-stratified findings, these results indicate that no coherent microbial signature emerged from stratification by *H. pylori* status, tumor stage, or lesion location, nor by co-existing type 2 diabetes. Confidence intervals around these effect sizes were wide in every case, and the subgroup sample sizes were modest; these analyses therefore establish that no difference was detected rather than that none exists. Within the limits of this power, the guild-level depletion observed in gastric cancer did not vary appreciably across the clinical strata examined (Figure 2b–d).

## 4. Discussion

In this cross-sectional study of 97 Korean adults, we characterized the fecal microbiota of patients with gastric cancer and examined whether co-existing type 2 diabetes further modified the GC-associated microbial signature. Patients with GC showed a distinct microbial composition relative to healthy controls, marked by depletion across the short-chain fatty acid-producing commensals, including *Subdoligranulum*, *Blautia*, *Anaerostipes*, *Faecalibacterium*, and *Bifidobacterium*, which collectively fell from 26.71% of the fecal community in controls to 6.55% in patients with GC, and enrichment of *Veillonella* and *Enterobacteriaceae*. Within the GC cohort, SCFA-producer abundance was lower in patients with co-existing T2D, but the difference did not reach statistical significance and the confidence interval remained wide. Permutational testing showed no community-level differences by T2D status, *H. pylori* status, tumor stage, or lesion location, despite substantial anthropometric differences across lesion sites.

Depletion of butyrate-producing commensals and expansion of oral and opportunistic taxa are well-documented features of the fecal community in gastric cancer, reproduced here and confirmed in pooled analysis of nine independent cohorts [4,17]. What has not been tested is whether this signature is modified by the metabolic state of the host. Type 2 diabetes is a plausible modifier: it is associated with depletion of the same butyrate-producing taxa [10,25], co-occurs with gastric cancer more often than chance would predict [19], and carries an excess risk localized to specific anatomical subsites [20]. Were metabolic comorbidity to amplify this depletion, SCFA-producer abundance would fall further in diabetic patients, and microbiome-based risk stratification would need to account for metabolic status. We tested that expectation directly, within a single cohort and against three established clinical variables and detected no such amplification.

The dysbiotic pattern in our GC cohort aligns with prior fecal microbiome studies. A recent meta-analysis integrating 394 stool samples across multiple GC cohorts reported enrichment of *Streptococcus*, *Escherichia*, and *Klebsiella* alongside depletion of butyrate producers including *Faecalibacterium* and *Roseburia* [4]. Our data reproduced this convergent signature, combining *Streptococcus* enrichment with depletion spanning eight SCFA-producing genera, among them *Bifidobacterium*, *Faecalibacterium*, *Blautia*, *Subdoligranulum*, and *Anaerostipes*. The *Streptococcus* enrichment is further supported by a multicenter study of 1043 patients across ten Chinese hospitals, in which *S. anginosus* and *S. constellatus* discriminated GC patients from controls [26], and by a 16S cohort of 116 GC patients and 88 controls from Shanxi Province [5] reporting the same depletion-and-expansion pattern. Convergent evidence also comes from the gastric mucosa: a meta-analysis of 825 biopsies from six independent cohorts, adjusted for age, sex, and *H. pylori* status, identified *Bifidobacterium* and *Blautia* among the taxa consistently depleted in GC relative to superficial gastritis, and characterized them as putative probiotics whose co-occurrence weakened progressively along the carcinogenic sequence [27].

The genera enriched in our GC cohort were predominantly of oral or opportunistic origin. *Streptococcus*, *Veillonella*, *Prevotella*, and *Fusobacterium* are oral commensals, and *Klebsiella*, *Enterobacter*, and *Citrobacter* are *Enterobacteriaceae*, together accounting for the marked *Proteobacteria* expansion observed at the phylum level. This pattern is not particular to our cohort: a meta-analysis of nine fecal 16S datasets comprising 405 patients with GC and 349 controls reported a pooled increase in *Proteobacteria* of 12.46 percentage points (95% CI 3.06 to 21.87) and a corresponding reduction in *Firmicutes*, closely matching the magnitude we observed [17]. The same analysis found no consistent difference in alpha diversity between patients and controls across eight studies, indicating that compositional shifts rather than diversity loss characterizes the fecal community in GC. Hypochlorhydria accompanying gastric atrophy removes the acid barrier that ordinarily limits transit of swallowed organisms, and more than half of the genera enriched in gastric cancer mucosa are typical oral inhabitants [27]. This relationship may not be unidirectional. *Streptococcus anginosus*, an oral organism enriched in gastric cancer mucosa, colonizes the murine stomach and induces chronic gastritis, parietal cell atrophy, metaplasia, and dysplasia, with the effect reproduced in germ-free animals and mediated by a surface adhesin engaging epithelial Annexin A2 [6]. Because parietal cells secrete gastric acid, colonization by such organisms and loss of the barrier that excludes them may be mutually reinforcing rather than sequential. These are experimental observations in mice, and our cross-sectional data cannot establish that this process operated in the present cohort; they do, however, provide a mechanism by which the enrichment we observed could be more than a passive consequence of disease.

The taxa lost from the community were not a random assortment. *Faecalibacterium*, *Subdoligranulum*, and *Anaerostipes* are terminal butyrate producers of clostridial clusters IV and XIVa [28], while *Bifidobacterium*, *Blautia*, *Dorea*, *Fusicatenibacter*, and *Lachnospira* generate the acetate and lactate that these organisms consume, a cross-feeding dependency that couples the two tiers metabolically [29]. This dependency is direct: *Faecalibacterium* generates butyrate through butyryl-CoA: acetate CoA-transferase, using extracellular acetate as substrate, and acetate cross-feeding from *Bifidobacterium* has been demonstrated experimentally [30]. Loss of both simultaneously implies a collapse of substrate supply as well as of terminal capacity and predicts a reduction in butyrate output disproportionate to the loss of any one genus. This has a predictable ecological consequence. Butyrate is the preferred substrate of colonocytes, and its oxidation through PPAR-γ-driven β-oxidation consumes epithelial oxygen and maintains the luminal anaerobiosis on which obligate fermenters depend; when butyrate supply falls, colonocyte metabolism shifts, oxygen diffuses into the lumen, and facultative anaerobes gain a respiratory advantage [31]. The concurrent enrichment of *Proteobacteria* in our cohort, rising from 1.84% in controls to 12.94% in patients and driven by *Klebsiella*, *Enterobacter*, and *Citrobacter*, is consistent with this sequence. On this reading the two dominant features of the GC-associated fecal community are not independent observations but successive stages of one process, in which loss of the fermentative guild creates the conditions for opportunistic expansion. This matters because butyrate is the functional endpoint through which these taxa act on the mucosa; it reinforces tight-junction integrity, suppresses NF-κB-driven IL-8 production [32], and promotes IL-10-producing regulatory T cells [33]. In diabetic mice, the *F. prausnitzii*-derived microbial anti-inflammatory molecule (MAM) restores intestinal barrier structure and ZO-1 expression under hyperglycemic conditions [34], and oral *F. prausnitzii* lowers fasting glucose, improves insulin resistance, and attenuates hepatic and adipose inflammation [12]. Loss of these functions removes an anti-inflammatory constraint from an already inflamed mucosa and may foster a tumor-permissive milieu [4]. These mechanistic links are drawn from experimental models and do not establish that reduced butyrate production occurred in this cohort, in which SCFAs were not measured.

Against this background, the further contraction of SCFA-producer abundance in patients with co-existing T2D did not reach significance, and we report it as a trend rather than an effect. The direction is nonetheless consistent with extensive literature: across 58 observational cohorts, *Faecalibacterium* is negatively associated with T2D and *F. prausnitzii* regarded as protective [10]; across 784 gut metagenomes analyzed with antidiabetic medication controlled, T2D carries a unified signature of butyrate-producer depletion [25]; and in adults spanning the dysglycemic spectrum, *F. prausnitzii* sits among the most highly connected members of an intercorrelated butyrate-producing network whose abundance tracks insulin sensitivity and circulating butyrate [9]. With 17 diabetic patients, our study was underpowered to resolve an increment of this size against the much larger shift already imposed by malignancy, and the question remains open rather than answered.

Stratification by *H. pylori* status, tumor stage and lesion location produced no detectable community-level differences and no change in SCFA-producer abundance, in contrast to the magnitude of the shift separating patients from controls. *H. pylori* seroprevalence in our cohort (47.9%) fell within the expected range for Korean adults [35]. Against an unchanged guild, *Faecalibacterium* alone was higher in infected patients, opposite in direction to the recovery reported after eradication [36]. This isolated genus-level difference did not survive correction and was not accompanied by shifts in the surrounding functionally linked taxa around. Tumor stage likewise produced no differences, which is biologically coherent given that the major gastric microbiome shifts occur early, during the transition to intraepithelial neoplasia, with limited divergence thereafter [37,38]; fecal profiles at later stages therefore reflect established tumor biology more than ongoing microbial reorganization. Lesion location produced the clearest dissociation between host phenotype and fecal microbiota. Upper-third (cardia) tumors arose more often in heavier male patients with higher BMI, in keeping with the established epidemiology of cardia GC, where male sex and obesity are principal risk factors [39,40,41], and mirroring the subsite-specific excess risk reported for T2D in pooled individual-participant data [20]. This dissociation is sharpened by evidence that the gastric mucosa does discriminate between subsites: cardia and non-cardia cancers carry significantly different mucosal community structures, with higher *Helicobacter* abundance and lower Shannon diversity in cardia cases [42]. Our fecal samples registered none of this, despite the marked anthropometric divergence across lesion groups within the same cohort. Subsite biology that is legible at the gastric mucosa therefore does not propagate to the distal compartment, which is consistent with stool reflecting the aggregate consequences of disease rather than its anatomical origin, and with site-specific microbial differences in GC being demonstrated more consistently in gastric mucosa than in fecal samples [27].

Taken together, these observations describe a single ecological process. The commensal fermentation network contracted across both its terminal butyrate producers and the acetate- and lactate-supplying taxa that sustain them, while oral and opportunistic organisms appeared in samples where they had previously been absent, yielding a community whose variable fraction was dominated by organisms contributing no butyrate. The same compositional signature has been reported in 455 fecal samples, with *Streptococcus* and *Veillonella* among the genera best discriminating patients from controls [43]. What our stratified analyses add is that this signature does not resolve further: no difference in SCFA-producer abundance was detected by *H. pylori* status, tumor stage, lesion location, nor co-existing T2D, despite marked differences in host anthropometry across lesion sites and in glucose and lipid profile between diabetic subgroups. Metabolic derangement was thus detectable in these patients yet left no fecal microbial trace, consistent with major gastric community shifts accruing early in the carcinogenic sequence [37,38] and with fecal SCFA-producer depletion marking established disease rather than tracking its subdivisions. A signature uniform across strata is a poor instrument for stratification but a serviceable one for detection, and its guild-level aggregate is a functional rather than taxonomic readout, less exposed to the population-specific genus-level variation noted above. Whether this depletion contributes to carcinogenesis or follows from it awaits prospective sampling across the precancerous cascade with direct quantification of fecal SCFAs.

Within controls, SCFA-producer abundance was positively rather than inversely associated with fasting glucose and hepatic enzymes. This direction is less anomalous than it first appears. Butyrate-producing taxa do not behave as a metabolically uniform group: in a scoping review of 45 observational studies, *Faecalibacterium* was inversely associated with glucose dysregulation while *Dorea* was directly associated, independently of medication and body measures [44], and in adults spanning the dysglycemic spectrum two butyrate-producing species showed adverse associations with insulin sensitivity, inversely correlated with the species conferring metabolic benefit [9]. In our cohort the positive associations were carried by *Subdoligranulum*, *Blautia*, and *Dorea*, with no association for *Faecalibacterium*, consistent with this heterogeneity. Aggregating genera into a single measure necessarily obscures such divergence, and these correlations, derived from 34 individuals across a narrow physiological range and uncorrected for multiple comparisons, should be treated as exploratory.

Several limitations should be considered. This was a cross-sectional study, conducted at a single clinical center, with controls recruited from two institutes, which precludes causal inference and limits generalizability beyond the Korean population. 16S rRNA gene sequencing resolves taxonomy rather than function, so SCFA production was inferred from the abundance of producing taxa rather than measured directly. Genus-level 16S assignment is also an imperfect quantitative tool for *Faecalibacterium* specifically, as the genus has been resolved into multiple species with sufficient sequence divergence to bias abundance estimates [30]. The diabetic subgroup was modest (*n* = 17), and the *H. pylori*, stage, and lesion location analyses were correspondingly underpowered; consistent with this, the GC-versus-control signature was robust to Benjamini–Hochberg correction, whereas the within-GC comparisons were not. The absence of a T2D-only comparison group also precluded separation of the microbiome signature attributable to T2D alone from that associated with gastric cancer and prevented formal assessment of a GC-by-T2D interaction. Subgroup analyses were accompanied by wide confidence intervals, and the absence of detected differences should not be interpreted as evidence of equivalence. Several exposures known to shape the gut microbiota require consideration. Antibiotics, probiotics, and acid-suppressive therapy within four weeks of sampling were exclusion criteria in both groups, removing the most immediate pharmacological sources of confounding; longer-term proton pump inhibitor exposure was not recorded. Metformin use was not documented. Metformin is known to alter the gut microbiome, including pathways related to SCFA production [25]; consequently, without medication data, we cannot determine its potential contribution to the observed T2D-associated pattern. Dietary intake was not assessed, and fermented-food consumption is high in Korean populations and a recognized determinant of commensal abundance. Notwithstanding these constraints, the study has several strengths. To our knowledge, it is the first to examine GC-associated fecal dysbiosis stratified by co-existing type 2 diabetes within a single cohort, allowing the microbial contributions of metabolic comorbidity and malignancy to be considered together rather than in isolation. Analyzing a defined panel of SCFA-producing genera as an aggregate reduced the influence of population-specific variation in individual genera, and false discovery rate control across multiple stratifications together with permutational testing of community structure supports the internal reliability of the findings.

Several questions follow. Whether the compositional depletion reported here translates into reduced luminal butyrate requires direct quantification of fecal short-chain fatty acids alongside sequencing, and shotgun metagenomics would measure butyrate-synthesis gene content rather than inferring it from genus-level assignment. Whether metabolic comorbidity modifies GC-associated dysbiosis remains open; resolving it will require cohorts stratified by metabolic status at the design stage, with medication exposure recorded prospectively. The aggregate measure used here was derived from taxa depleted in this cohort, and its behavior in independent populations, and across the precancerous cascade rather than at a single time point, will determine whether it holds beyond description.

## 5. Conclusions

Gastric cancer in this Korean cohort was accompanied by depletion extending across the fecal short-chain fatty acid-producing guild rather than confined to individual genera, with concurrent expansion of oral and opportunistic taxa. Analyzing these genera as an aggregate captured a change that single-taxon comparisons describe only in part and may provide a more stable summary than genus-level fingerprints, which vary between populations. Stratification by co-existing type 2 diabetes, *Helicobacter pylori* status, tumor stage, and lesion location detected no statistically significant modifications of this signature; subgroup sizes were modest, and these analyses establish non-detection rather than equivalence. In this cohort, the fecal microbial signature distinguished gastric cancer from controls more clearly than it differentiated the examined clinical or metabolic subgroups. Establishing whether the taxonomic depletion reported here corresponds to reduced luminal butyrate, and whether it precedes or follows malignancy, will require prospective sampling across the precancerous cascade with direct quantification of short-chain fatty acids.

## Figures and Tables

**Table 1:** Baseline demographic and biochemical characteristics of the study cohort.

Variable		Reference Range	Control (*n* = 34)	GC (*n* = 63)	*p*-Value
Sex, *n* (%)	Male		18 (52.9)	42 (66.7)	0.184
	Female		16 (47.1)	21 (33.3)	
Age (years)			50.85 ± 3.80	62.54 ± 12.59	**<0.001**
Weight (kg)			67.54 ± 12.14	66.45 ± 12.19	0.675
BMI (kg/m^2^)			24.44 ± 3.37	24.44 ± 3.44	0.996
Glucose (mg/dL)		70–100	114.63 ± 33.33	124.37 ± 40.78	0.236
Total cholesterol (mg/dL)		<200	170.29 ± 37.12	181.10 ± 45.00	0.234
AST (IU/L)		M 0–40/F 0–32	22.30 ± 15.93	27.63 ± 14.48	0.098
ALT (IU/L)		M 0–41/F 0–33	10.47 ± 7.43	25.52 ± 18.04	**<0.001**

Values are presented as mean ± standard deviation unless otherwise indicated. Sex is presented as *n* (%). Bold *p*-values indicate statistical significance (*p* < 0.05). ALT, alanine aminotransferase; AST, aspartate aminotransferase; BMI, body mass index; GC, gastric cancer.

**Table 2:** Differentially abundant gut microbial taxa between healthy controls and patients with gastric cancer.

Taxon	Control (*n* = 34)	GC (*n* = 63)	*p*-Value	Cliff’s δ (95% CI)
**Phylum level**				
*Fusobacteria*	0.03 ± 0.16	0.33 ± 1.73	**<0.001**	−0.75 (−0.90, −0.59)
*Proteobacteria*	1.84 ± 3.01	12.94 ± 16.00	**<0.001**	−0.63 (−0.78, −0.44)
*Verrucomicrobia*	0.08 ± 0.16	0.98 ± 3.64	**<0.001**	−0.47 (−0.67, −0.23)
*Synergistetes*	0.02 ± 0.05	0.02 ± 0.04	**0.005**	−0.35 (−0.59, −0.10)
*Firmicutes*	66.04 ± 20.63	54.33 ± 19.09	**0.006**	+0.34 (+0.09, +0.56)
*Actinobacteria*	7.38 ± 8.29	4.10 ± 7.78	**0.048**	+0.24 (−0.02, +0.50)
F/B ratio	18.67 ± 35.45	6.92 ± 20.15	0.080	+0.22 (−0.04, +0.47)
**Genus Level**				
**Depleted in GC**				
*Subdoligranulum*	2.16 ± 3.37	0.09 ± 0.20	**<0.001**	+0.79 (+0.64, +0.90)
*Anaerostipes*	1.45 ± 1.71	0.12 ± 0.29	**<0.001**	+0.77 (+0.62, +0.89)
*Blautia*	7.03 ± 7.58	0.49 ± 0.68	**<0.001**	+0.72 (+0.54, +0.87)
*Faecalibacterium*	8.03 ± 6.56	3.08 ± 4.02	**<0.001**	+0.57 (+0.38, +0.75)
*Bifidobacterium*	4.87 ± 6.42	1.70 ± 7.05	**<0.001**	+0.57 (+0.36, +0.75)
*Fusicatenibacter*	0.89 ± 0.94	0.29 ± 0.66	**<0.001**	+0.54 (+0.32, +0.75)
*Lachnospira*	0.13 ± 0.27	0.07 ± 0.37	**<0.001**	+0.49 (+0.27, +0.70)
*Dorea*	2.15 ± 2.50	0.70 ± 1.11	**0.003**	+0.37 (+0.12, +0.58)
SCFA-producer abundance	26.71 ± 16.92	6.55 ± 8.25	**<0.001**	+0.76 (+0.61, +0.89)
**Enriched in GC**				
*Eubacterium*	0.23 ± 0.48	4.51 ± 7.94	**<0.001**	−0.83 (−0.93, −0.70)
*Veillonella*	0.26 ± 0.75	1.85 ± 3.33	**<0.001**	−0.72 (−0.87, −0.54)
*Klebsiella*	0.08 ± 0.23	1.25 ± 3.52	**<0.001**	−0.64 (−0.82, −0.45)
*Enterobacter*	0.05 ± 0.20	0.91 ± 2.86	**<0.001**	−0.63 (−0.80, −0.44)
*Streptococcus*	2.12 ± 5.59	10.29 ± 14.03	**<0.001**	−0.60 (−0.77, −0.41)
*Fusobacterium*	0.03 ± 0.16	0.35 ± 1.81	**<0.001**	−0.57 (−0.73, −0.38)
*Prevotella*	7.39 ± 13.55	11.16 ± 18.04	**0.012**	−0.31 (−0.56, −0.06)

Values are mean ± standard deviation relative abundance (%). Comparisons by Mann–Whitney U test; only taxa differing significantly (*p* < 0.05) are shown. Cliff’s delta is reported with bootstrap 95% confidence intervals; positive values indicate higher abundance in controls. SCFA-producer abundance is defined in Section 2.8. Full taxonomic data, FDR-adjusted *q*-values, and CLR-based results are provided in Supplementary Table S1. F/B, *Firmicutes*-to-*Bacteroidetes* ratio. Bold values indicate *p* < 0.05.

**Table 3:** Clinical characteristics and SCFA-producer abundance in patients with gastric cancer stratified by type 2 diabetes status.

Variable		Non-Diabetes (*n* = 46)	Diabetes (*n* = 17)	*p*-Value	Cliff’s δ (95% CI)
**Demographic and biochemical characteristics**					
Sex, *n* (%)	Male	30 (65.22)	12 (70.59)	0.688	
	Female	16 (34.78)	5 (29.41)		
Age (years)		60.80 ± 12.96	67.24 ± 10.44	0.072	
Weight (kg)		66.80 ± 12.11	65.50 ± 12.74	0.712	
BMI (kg/m^2^)		24.52 ± 3.48	24.24 ± 3.42	0.772	
Glucose (mg/dL)		117.24 ± 30.45	143.65 ± 57.40	0.087	
Total cholesterol (mg/dL)		188.30 ± 45.06	161.59 ± 39.77	**0.035**	
AST (IU/L)		25.76 ± 13.22	32.71 ± 16.84	0.091	
ALT (IU/L)		24.93 ± 19.31	27.12 ± 14.45	0.673	
**SCFA-producing genera (%)**					
*Faecalibacterium*		3.56 ± 4.52	1.77 ± 1.65	0.373	+0.15 (−0.14, +0.43)
*Blautia*		0.49 ± 0.70	0.49 ± 0.64	0.975	+0.01 (−0.34, +0.33)
*Subdoligranulum*		0.09 ± 0.19	0.12 ± 0.22	0.714	−0.06 (−0.38, +0.28)
*Anaerostipes*		0.12 ± 0.27	0.11 ± 0.36	0.122	+0.26 (−0.06, +0.56)
*Fusicatenibacter*		0.30 ± 0.74	0.25 ± 0.35	0.727	−0.06 (−0.38, +0.26)
*Dorea*		0.69 ± 1.02	0.73 ± 1.35	0.988	−0.00 (−0.30, +0.31)
*Lachnospira*		0.02 ± 0.05	0.20 ± 0.70	0.086	−0.28 (−0.57, +0.03)
*Bifidobacterium*		2.18 ± 8.22	0.42 ± 0.62	0.303	+0.17 (−0.14, +0.48)
SCFA-producer abundance		7.45 ± 9.24	4.10 ± 3.87	0.152	+0.24 (−0.09, +0.52)
**Other differentially abundant taxa (%)**					
*Enterobacter*		1.20 ± 3.30	0.12 ± 0.20	**0.029**	+0.36 (+0.04, +0.65)
*Veillonella*		2.03 ± 3.23	1.36 ± 3.64	**0.027**	+0.37 (+0.03, +0.67)

Values are presented as mean ± standard deviation unless otherwise indicated. Microbial taxa are shown as relative abundance (%). SCFA-producer abundance is defined in Section 2.8. Clinical variables were compared by independent two-sample *t*-tests and microbial taxa by Mann–Whitney *U* tests. No genus-level comparison survived Benjamini–Hochberg FDR correction; full taxonomic data and *q*-values are provided in Supplementary Table S2. Cliff’s δ is reported with bootstrap 95% confidence intervals for microbial comparisons; positive values indicate greater abundance in the first group relative to the second group. Bold *p*-values indicate statistical significance (*p* < 0.05). ALT, alanine aminotransferase; AST, aspartate aminotransferase; BMI, body mass index; SCFA, short-chain fatty acid.

**Table 4:** Clinical characteristics and gut microbial composition in patients with gastric cancer stratified by *Helicobacter pylori* infection status.

Variable		*H. pylori* Negative (*n* = 25)	*H. pylori* Positive (*n* = 23)	*p*-Value	Cliff’s δ (95% CI)
**Clinical variables**					
Sex, *n* (%)	Male	16 (64.0)	15 (65.2)	0.930	
	Female	9 (36.0)	8 (34.78)		
Age (years)		66.68 ± 11.87	61.00 ± 11.97	0.106	
Weight (kg)		64.33 ± 11.37	64.59 ± 11.36	0.939	
BMI (kg/m^2^)		23.92 ± 3.09	24.13 ± 3.00	0.812	
Glucose (mg/dL)		132.60 ± 47.69	117.83 ± 41.32	0.259	
Cholesterol (mg/dL)		174.56 ± 41.67	191.09 ± 45.23	0.194	
AST (IU/L)		30.72 ± 18.35	28.13 ± 13.12	0.580	
ALT (IU/L)		26.60 ± 18.22	27.52 ± 20.83	0.871	
**Gut microbial composition (% relative abundance, mean ± SD)**					
**Phylum level**					
*Bacteroidetes*		27.37 ± 17.94	27.02 ± 23.90	0.584	+0.09 (−0.25, +0.44)
*Firmicutes*		50.36 ± 19.19	56.37 ± 20.25	0.176	−0.23 (−0.53, +0.10)
*Proteobacteria*		16.12 ± 18.80	11.59 ± 12.58	0.369	+0.15 (−0.18, +0.46)
*Actinobacteria*		4.11 ± 10.99	3.16 ± 3.80	0.584	−0.09 (−0.42, +0.26)
F/B ratio		9.90 ± 31.15	6.06 ± 7.91	0.392	−0.15 (−0.47, +0.17)
**Genus level**					
*Faecalibacterium*		1.53 ± 1.51	4.06 ± 4.95	**0.039**	−0.35 (−0.63, −0.03)
*Bifidobacterium*		2.82 ± 11.01	0.38 ± 0.57	0.342	+0.16 (−0.17, +0.48)
*Streptococcus*		9.01 ± 11.92	14.65 ± 18.05	0.483	−0.12 (−0.45, +0.22)
*Enterobacter*		1.32 ± 4.00	0.88 ± 2.22	0.269	+0.19 (−0.16, +0.53)
*Megamonas*		0.62 ± 1.73	0.41 ± 0.99	**0.050**	−0.33 (−0.62, +0.00)
*Gemella*		0.05 ± 0.09	0.19 ± 0.32	**0.058**	−0.32 (−0.62, −0.00)
SCFA-producer abundance		5.89 ± 10.90	6.32 ± 6.29	0.332	**−0.17 (−0.50, +0.16)**

Values are presented as mean ± standard deviation unless otherwise indicated. Sex is presented as *n* (%). Clinical variables were compared by independent two-sample *t*-tests and microbial taxa by Mann–Whitney *U* tests. Cliff’s δ is reported with bootstrap 95% confidence intervals for microbial comparisons; positive values indicate greater abundance in the first group relative to the second group. Bold *p*-values indicate statistical significance (*p* < 0.05). ALT, alanine aminotransferase; AST, aspartate aminotransferase; BMI, body mass index; F/B, *Firmicutes*-to-*Bacteroidetes*.

**Table 5:** Clinical characteristics and gut microbial composition in patients with gastric cancer stratified by tumor stage.

Variable		Stage I (*n* = 43)	≥Stage II (*n* = 19)	*p*-Value	Cliff’s δ (95% CI)
**Clinical variables**					
Sex, *n* (%)	Male	29 (67.44)	13 (68.42)	0.939	
	Female	14 (32.56)	6 (31.58)		
Age (years)		64.74 ± 11.83	59.21 ± 11.85	0.095	
Weight (kg)		67.07 ± 12.52	63.63 ± 9.83	0.294	
BMI (kg/m^2^)		24.49 ± 3.49	23.89 ± 2.81	0.516	
Glucose (mg/dL)		128.93 ± 46.93	115.16 ± 20.59	0.114	
Cholesterol (mg/dL)		182.65 ± 48.32	176.74 ± 38.56	0.640	
AST (IU/L)		30.21 ± 16.06	22.26 ± 8.35	**0.047**	
ALT (IU/L)		28.05 ± 19.98	20.37 ± 11.83	0.125	
**Gut microbial composition (% relative abundance, mean ± SD)**					
**Phylum level**					
*Bacteroidetes*		27.33 ± 21.09	24.97 ± 14.83	0.885	+0.02 (−0.28, +0.31)
*Firmicutes*		54.02 ± 19.99	56.06 ± 17.28	0.813	−0.04 (−0.34, +0.26)
*Proteobacteria*		13.23 ± 17.17	12.04 ± 13.83	0.897	+0.02 (−0.29, +0.33)
*Actinobacteria*		4.45 ± 9.12	3.23 ± 3.63	0.861	−0.03 (−0.37, +0.29)
F/B ratio		8.48 ± 24.23	3.70 ± 3.15	0.945	−0.01 (−0.31, +0.29)
**Genus level**					
*Faecalibacterium*		2.61 ± 3.49	4.28 ± 4.98	0.096	−0.27 (−0.57, +0.06)
*Bifidobacterium*		2.11 ± 8.49	0.60 ± 0.95	0.423	+0.13 (−0.18, +0.45)
*Streptococcus*		10.47 ± 13.94	10.36 ± 14.83	0.807	+0.04 (−0.27, +0.39)
*Enterobacter*		0.96 ± 3.22	0.82 ± 1.96	0.288	+0.17 (−0.14, +0.49)
*Megamonas*		0.78 ± 1.81	0.16 ± 0.54	0.945	+0.01 (−0.29, +0.30)
*Gemella*		0.09 ± 0.21	0.11 ± 0.23	0.305	−0.17 (−0.45, +0.12)
SCFA-producer abundance		6.40 ± 9.21	6.88 ± 6.03	0.205	**−0.20 (−0.48, +0.09)**

Values are presented as mean ± standard deviation unless otherwise indicated. Sex is presented as *n* (%). Clinical variables were compared by independent two-sample t-tests and microbial taxa by Mann–Whitney *U* tests. Cliff’s δ is reported with bootstrap 95% confidence intervals for microbial comparisons; positive values indicate greater abundance in the first group relative to the second group. Bold *p*-values indicate statistical significance (*p* < 0.05). ALT, alanine aminotransferase; AST, aspartate aminotransferase; BMI, body mass index; F/B, *Firmicutes*-to-*Bacteroidetes*.

**Table 6:** Clinical characteristics and gut microbial composition in patients with gastric cancer stratified by primary tumor lesion location.

Variable		Upper (*n* = 16)	Middle (*n* = 18)	Lower (*n* = 25)	*p*-Value	ε^2^ (95% CI)
**Clinical variables**						
Sex, *n* (%)	Male	13 (81.25)	14 (77.78)	12 (48.00)	**0.041**	
	Female	3 (18.75)	4 (22.22)	13 (52.00)		
Age (years)		58.38 ± 14.71	63.61 ± 9.68	65.56 ± 11.90	0.184	
Weight (kg)		74.93 ± 10.78 ^b^	65.43 ± 10.75 ^a^	61.75 ± 10.82 ^a^	**0.001**	
BMI (kg/m^2^)		26.44 ± 3.81 ^b^	24.06 ± 2.84 ^a^	23.72 ± 2.42 ^a^	**0.016**	
Glucose (mg/dL)		123.50 ± 34.10	134.44 ± 56.69	116.80 ± 31.79	0.393	
Cholesterol (mg/dL)		182.88 ± 38.80	183.28 ± 43.97	183.28 ± 51.12	1.000	
AST (IU/L)		25.06 ± 15.85	31.67 ± 8.28	27.44 ± 17.24	0.413	
ALT (IU/L)		26.50 ± 21.65	31.00 ± 13.73	23.00 ± 18.81	0.374	
**Gut microbial composition (% relative abundance, mean ± SD)**						
**Phylum level**						
*Bacteroidetes*		31.00 ± 21.09	25.56 ± 22.58	25.46 ± 16.69	0.659	0.014 (0.001, 0.173)
*Firmicutes*		57.90 ± 19.37	55.92 ± 22.74	51.30 ± 15.78	0.424	0.030 (0.002, 0.200)
*Proteobacteria*		7.25 ± 8.93	12.86 ± 15.58	15.55 ± 19.34	0.466	0.026 (0.002, 0.182)
*Actinobacteria*		2.76 ± 1.96	3.80 ± 5.23	5.34 ± 11.45	0.918	0.003 (0.001, 0.138)
F/B ratio		5.48 ± 8.54	5.20 ± 6.52	9.64 ± 30.96	0.680	0.013 (0.001, 0.167)
**Genus level**						
*Faecalibacterium*		3.92 ± 3.82	2.03 ± 1.70	3.72 ± 5.27	0.318	0.040 (0.003, 0.204)
*Bifidobacterium*		0.56 ± 0.94	0.36 ± 0.35	3.37 ± 11.05	0.340	0.037 (0.002, 0.234)
*Streptococcus*		13.79 ± 15.34	12.30 ± 16.73	7.92 ± 11.56	0.554	0.020 (0.002, 0.182)
*Enterobacter*		0.21 ± 0.28	1.14 ± 2.78	1.22 ± 3.86	0.982	0.001 (0.001, 0.119)
*Megamonas*		0.73 ± 1.58	0.31 ± 0.73	0.77 ± 2.02	0.972	0.001 (0.001, 0.140)
*Gemella*		0.18 ± 0.33	0.10 ± 0.21	0.04 ± 0.10	0.124	0.072 (0.006, 0.267)
SCFA-producer abundance		6.53 ± 4.41	3.94 ± 3.87	8.97 ± 11.81	0.125	**0.072 (0.009, 0.265)**

Values are presented as mean ± standard deviation unless otherwise indicated. Sex is presented as *n* (%). Clinical variables were compared by one-way ANOVA and microbial taxa by Kruskal–Wallis tests. Different superscript letters (^a^, ^b^) indicate statistically significant pairwise differences between groups (post hoc test). Effect sizes for three-group comparisons are reported as epsilon-squared (ε^2^) with bootstrap 95% confidence intervals. Bold *p*-values indicate statistical significance (*p* < 0.05). ALT, alanine aminotransferase; AST, aspartate aminotransferase; BMI, body mass index; F/B, *Firmicutes*-to-*Bacteroidetes*.

**Table 7:** Community-level comparison of fecal microbiota composition by PERMANOVA.

Comparison	Pseudo-*F*	*R* ^2^	*p*-Value	PERMDISP *p*
Control vs. GC	9.20	0.088	**0.001**	0.297
T2D status (within GC)	1.07	0.017	0.358	0.429
*H. pylori* status	0.85	0.018	0.605	0.366
Tumor stage	0.69	0.011	0.840	0.152
Lesion location	1.18	0.040	0.209	0.433

PERMANOVA was performed on Bray–Curtis dissimilarity matrices with 999 permutations. *R*^2^ indicates the proportion of variation in community composition explained by the grouping variable. PERMDISP tests homogeneity of multivariate dispersion between groups. Bold *p*-values indicate statistical significance (*p* < 0.05). GC, gastric cancer; T2D, type 2 diabetes.

## Data Availability

The phylum- and genus-level relative abundance data and de-identified sample metadata supporting the findings of this study are openly available in Zenodo at https://doi.org/10.5281/zenodo.22885588. Raw sequence files were not retained and are therefore unavailable.

## Supplementary Materials

The following supporting information can be downloaded at: https://www.mdpi.com/article/10.3390/metabo16100738/s1, Table S1: Full phylum- and genus-level relative abundance comparisons between healthy controls and patients with gastric cancer, with Benjamini–Hochberg FDR-adjusted *q*-values; Table S2: Full phylum- and genus-level relative abundance comparisons between non-diabetic and diabetic patients with gastric cancer, with Benjamini–Hochberg FDR-adjusted *q*-values; Table S3: Spearman correlations between SCFA-producer abundance and metabolic variables; Table S4: Full phylum- and genus-level relative abundance comparisons in patients with gastric cancer stratified by *Helicobacter pylori* infection status; Table S5: Full phylum- and genus-level relative abundance comparisons in patients with gastric cancer stratified by tumor stage; Table S6: Full phylum- and genus-level relative abundance comparisons in patients with gastric cancer stratified by primary tumor lesion location; Figure S1: Alpha and beta diversity of fecal microbiota in patients with gastric cancer stratified by *Helicobacter pylori* infection status; Figure S2: Alpha and beta diversity of fecal microbiota in patients with gastric cancer stratified by tumor stage; Figure S3: Alpha and beta diversity of fecal microbiota in patients with gastric cancer stratified by primary tumor lesion location.

## Abbreviations

The following abbreviations are used in this manuscript:

ADA	American Diabetes Association
AJCC	American Joint Committee on Cancer
ALT	Alanine aminotransferase
ANOVA	Analysis of variance
AST	Aspartate aminotransferase
BMI	Body mass index
CT	Computed tomography
ELISA	Enzyme-linked immunosorbent assay
F/B	*Firmicutes*-to-*Bacteroidetes* ratio
FDR	False discovery rate
GC	Gastric cancer
HbA1c	Glycated hemoglobin
IgG	Immunoglobulin G
MRI	Magnetic resonance imaging
NMDS	Non-metric multidimensional scaling
OTU	Operational taxonomic unit
P/B	*Prevotella*-to-*Bacteroides* ratio
PCoA	Principal coordinates analysis
PET	Positron emission tomography
SCFA	Short-chain fatty acid
T2D	Type 2 diabetes
TNM	Tumor–Node–Metastasis (staging system)
UICC	Union for International Cancer Control
