# Supplementary material for: Depletion of Short-Chain Fatty Acid-Producing Commensals in Gastric Cancer Is Not Detectably Modified by Type 2 Diabetes or Tumor Characteristics
Source: Metabolites. 2026 Sep 30;16(10):738. doi: 10.3390/metabo16100738 (PMC13707191; doi:10.3390/metabo16100738)
Supplement: Supplementary file 1 [file metabolites-16-00738-s001.zip › metabolites-4546963-supplementary.pdf]

Supplementary Materials

Depletion of Short-Chain Fatty Acid-Producing Commensals in Gastric Cancer Is Not Detectably Modified by Type 2 Diabetes or Tumor Characteristics

Da-Hye Gu [1], Amara Zulfiqar [2], Sang-Ho Jeong [3], Hyun-Hi Kang [4], Hyun-Young Joo [4] and Jung-Eun Yim [1,2,*]

Supplementary Table S1. Full phylum- and genus-level relative abundance comparisons between healthy controls and patients with gastric cancer, with Benjamini–Hochberg false discovery rate-adjusted *q*-values

Variables	Control (n = 34)	GC (n = 63)	*p*-value	*q* (BH)	*q (CLR)*
**Phylum**					
Bacteroidetes	24.23±23.25	26.84±19.23	0.255	0.312	0.029
Firmicutes	66.04±20.63	54.33 ± 19.09	**0.006**	**0.012**	**<0.001**
Proteobacteria	1.84±3.01	12.94±16.00	**<0.001**	**<0.001**	**0.022**
Actinobacteria	7.38±8.29	4.10±7.78	**0.048**	**0.088**	**<0.001**
Verrucomicrobia	0.08±0.16	0.98±3.64	**<0.001**	**<0.001**	**0.008**
Fusobacteria	0.03±0.16	0.33±1.73	**<0.001**	**<0.001**	**<0.001**
Euryarchaeota	0.11±0.44	0.05±0.18	0.476	0.476	0.005
Synergistetes	0.02±0.05	0.02±0.04	**0.005**	**0.012**	**0.070**
Tenericutes	0.05±0.13	0.01±0.03	0.156	0.215	0.973
Lentisphaerae	0.00±0.00	0.01±0.03	0.340	0.374	**<0.001**
F/B ratio	18.67±35.45	6.92±20.15	0.080	0.126	—
**Genus**					
Bacteroides	12.33±16.30	9.80±12.35	0.961	0.961	0.093
Prevotella	7.39±13.55	11.16±18.04	**0.012**	0.024	0.259
Streptococcus	2.12±5.59	10.29±14.03	**<0.001**	**<0.001**	**0.002**
Lactobacillus	3.88±7.05	4.17±9.85	0.302	0.384	0.967
Lactococcus	0.01±0.03	0.09±0.31	0.715	0.736	0.380
Eubacterium	0.23±0.48	4.51±7.94	**<0.001**	**<0.001**	**<0.001**
Faecalibacterium	8.03±6.56	3.08±4.02	**<0.001**	**<0.001**	**<0.001**
Bifidobacterium	4.87±6.42	1.70±7.05	**<0.001**	**<0.001**	**<0.001**
Ruminococcus	1.54±2.94	1.13±2.95	**0.300**	**0.384**	**0.098**
Enterobacter	0.05±0.20	0.91±2.86	**<0.001**	**<0.001**	**<0.001**
Enterococcus	0.70±2.66	0.76±3.15	0.123	0.176	0.690
Fusobacterium	0.03±0.16	0.35±1.81	**<0.001**	**<0.00**	**<0.001**
Veillonella	0.26±0.75	1.85±3.33	**<0.001**	**<0.001**	**<0.001**
Haemophilus	0.53±2.23	1.00±3.44	0.004	0.009	0.068
Paraprevotella	0.44±0.95	0.26±0.41	0.649	0.699	0.720
Catenibacterium	1.55±2.81	0.26±0.63	0.293	0.384	0.155
Salmonella	0.00±0.01	0.11±0.32	**<0.001**	0.001	**0.017**
Dorea	2.15±2.50	0.70±1.11	**0.003**	**0.007**	**<0.001**
Citrobacter	0.04±0.15	1.09±6.39	**<0.001**	**<0.001**	**0.010**
Gemella	0.01±0.03	0.09±0.22	**0.002**	**0.005**	0.078
Megamonas	0.41±2.03	0.58±1.55	0.084	0.128	0.380
Blautia	7.03±7.58	0.49±0.68	**<0.001**	**<0.001**	**<0.001**
Subdoligranulum	2.16±3.37	0.09±0.20	**<0.001**	**<0.001**	**<0.001**
Anaerostipes	1.45±1.71	0.12±0.29	**<0.001**	**<0.001**	**<0.001**
Fusicatenibacter	0.89±0.94	0.29±0.66	**<0.001**	**<0.001**	**<0.001**
Lachnospira	0.13±0.27	0.07±0.37	**<0.001**	**<0.001**	**<0.001**
Roseburia	1.41±1.95	1.43±1.60	0.494	0.565	0.230
Coprococcus	0.31±0.46	0.32±0.40	0.403	0.495	0.818

Oscillibacter	1.61±2.89	1.93±3.48	0.489	0.565	0.806
Butyricimonas	0.33±1.38	0.39±0.82	0.018	0.034	0.169
Odoribacter	0.05±0.08	0.05±0.07	0.942	0.955	0.311
Phascolarctobacterium	0.76±2.15	0.35±0.88	0.695	0.726	0.788
Megasphaera	0.26±0.72	0.13±0.39	0.613	0.670	0.677
Pseudobutyrivibrio	0.03±0.08	0.03±0.06	0.023	0.041	0.117
Akkermansia	0.08±0.17	1.07±3.90	0.005	**0.010**	0.06
Klebsiella	0.08±0.23	1.25±3.52	**<0.001**	**<0.001**	**<0.001**
Atopobium	0.00±0.00	0.24±1.04	**<0.001**	**<0.001**	**<0.001**
P/B ratio	3.27±7.51	8.12±29.48	0.103	0.151	—

Values are presented as mean ± standard deviation relative abundance (%). *p*-values are from Mann-Whitney U tests. *q* (BH) = Benjamini–Hochberg false discovery rate-adjusted q-value, computed separately within the phylum-level and genus-level families of tests. *q* (CLR) = Benjamini–Hochberg-adjusted p-value from Mann-Whitney *U* tests on centered log-ratio-transformed abundances, restricted to taxa present in at least 10% of samples; ratios were not included in this analysis. Bold values indicate statistical significance (p<0.05). F/B, *Firmicutes*-to-*Bacteroidetes* ratio; GC, gastric cancer; P/B, *Prevotella*-to-*Bacteroides* ratio.

Supplementary Table S2. Full phylum- and genus-level relative abundance comparisons between non-diabetic and diabetic patients with gastric cancer, with Benjamini–Hochberg false discovery rate-adjusted *q*-values

Variables	Non-diabetes (n = 46)	Diabetes (n = 17)	*p*-value	*q* (BH)	*q (CLR)*
**Phylum**					
Bacteroidetes	24.64±16.58	32.79±24.68	0.310	0.427	0.656
Firmicutes	54.25±18.25	54.53±21.78	0.541	0.615	0.606
Proteobacteria	14.15±17.13	9.68±12.31	0.268	0.421	0.358
Actinobacteria	4.97±8.94	1.76±1.38	0.067	0.122	0.152
Verrucomicrobia	1.11±4.09	0.63±2.05	**0.033**	0.122	0.139
Fusobacteria	0.37±1.97	0.24±0.86	0.056	0.122	0.139
Euryarchaeota	0.04±0.20	0.06±0.10	**0.005**	**0.026**	0.139
Synergistetes	0.01±0.02	0.05±0.06	0.064	0.122	0.152
Tenericutes	0.01±0.02	0.02±0.04	0.593	0.615	0.439
Lentisphaerae	0.00±0.03	0.01±0.02	**<0.001**	**0.007**	0.139
F/B ratio	8.24±23.45	3.35±3.01	0.615	0.615	—
**Genus**					
Bacteroides	9.37±11.33	10.95±15.09	0.551	0.736	0.777
Prevotella	10.03±15.14	14.20±24.55	0.798	0.890	0.796
Streptococcus	10.67±12.47	9.27±18.01	0.079	0.325	0.373
Lactobacillus	4.26±8.47	3.94±13.21	0.116	0.387	0.373
Lactococcus	0.10±0.35	0.05±0.16	0.704	0.864	0.961
Eubacterium	4.56±8.78	4.35±5.21	0.434	0.717	0.565
Faecalibacterium	3.56±4.52	1.77±1.65	0.373	0.672	0.804
Bifidobacterium	2.18±8.22	0.42±0.62	0.303	0.576	0.593
Ruminococcus	0.54±0.73	2.72±5.33	0.236	0.527	0.499
Enterobacter	1.20±3.30	0.12±0.20	**0.029**	0.305	0.153
Enterococcus	1.02±3.66	0.07±0.09	0.556	0.736	0.842
Fusobacterium	0.39±2.06	0.24±0.87	0.139	0.407	0.382
Veillonella	2.03±3.23	1.36±3.64	**0.027**	0.305	0.334
Haemophilus	1.30±3.98	0.20±0.53	0.058	0.325	0.334
Paraprevotella	0.23±0.41	0.33±0.41	0.278	0.567	0.628
Catenibacterium	0.30±0.69	0.16±0.41	0.283	0.567	0.593
Salmonella	0.13±0.37	0.03±0.05	0.820	0.890	0.988
Dorea	0.69±1.02	0.73±1.35	0.988	0.988	0.961

Variables	Non-diabetes (n = 46)	Diabetes (n = 17)	*p*-value	*q* (BH)	*q (CLR)*
Citrobacter	1.47±7.46	0.05±0.07	0.226	0.527	0.499
Gemella	0.10±0.21	0.09±0.23	0.070	0.325	0.373
Megamonas	0.72±1.73	0.20±0.80	0.225	0.527	0.508
Blautia	0.49±0.70	0.49±0.64	0.975	0.988	0.961
Subdoligranulum	0.09±0.19	0.12±0.22	0.714	0.864	0.830
Anaerostipes	0.12±0.27	0.11±0.36	0.122	0.387	0.334
Fusicatenibacter	0.30±0.74	0.25±0.35	0.727	0.864	0.852
Lachnospira	0.02±0.05	0.20±0.70	0.086	0.325	0.263
Roseburia	1.41±1.60	1.48±1.64	0.926	0.977	0.848
Coprococcus	0.31±0.35	0.34±0.51	0.804	0.890	0.985
Oscillibacter	1.70±2.85	2.57±4.85	0.180	0.490	0.382
Butyricimonas	0.32±0.75	0.58±0.99	**0.032**	0.305	0.190
Odoribacter	0.03±0.05	0.10±0.09	**0.002**	0.093	0.153
Phascolarctobacterium	0.32±0.82	0.44±1.06	0.479	0.736	0.593
Megasphaera	0.06±0.13	0.31±0.71	0.508	0.736	0.834
Pseudobutyrivibrio	0.03±0.05	0.05±0.08	0.389	0.672	0.525
Akkermansia	1.22±4.38	0.66±2.17	0.053	0.325	0.304
Klebsiella	1.02±3.10	1.88±4.51	0.500	0.736	0.796
Atopobium	0.31±1.22	0.06±0.12	0.080	0.325	0.334
P/B ratio	5.65±18.57	14.81±48.29	0.561	0.736	—

Values are presented as mean ± standard deviation relative abundance (%). *p*-values are from Mann-Whitney U tests. *q* (BH) = Benjamini–Hochberg false discovery rate-adjusted q-value, computed separately within the phylum-level and genus-level families of tests. *q* (CLR) = Benjamini–Hochberg-adjusted p-value from Mann-Whitney U tests on centered log-ratio-transformed abundances, restricted to taxa present in at least 10% of samples; ratios were not included in this analysis. Bold values indicate statistical significance ($p < 0.05$); no comparison remained significant after false discovery rate correction. F/B, *Firmicutes*-to-*Bacteroidetes* ratio; GC, gastric cancer; P/B, *Prevotella*-to-*Bacteroides* ratio.

Supplementary Table S3. Spearman correlations between SCFA-producer abundance and metabolic variables, assessed separately within healthy controls and patients with gastric cancer.

Variable	Controls (n = 34)		Gastric cancer (n = 62)	
	rho	*p*-value	*rho*	*p*-value
BMI (kg/m²)	+0.332	0.055	+0.143	0.267
Fasting glucose (mg/dL)	+0.401	**0.019**	+0.100	0.437
Total cholesterol (mg/dL)	+0.187	0.290	+0.163	0.205
AST (IU/L)	+0.499	**0.003**	-0.220	0.086
ALT (IU/L)	+0.422	**0.013**	-0.081	0.531

Values are Spearman rank correlation coefficients (rho) with corresponding p-values, calculated separately within each group. SCFA-producer abundance is the summed relative abundance of the SCFA-producing genera defined in Section 2.8. Bold values indicate statistical significance ($p<0.05$); *p*-values are uncorrected for multiple comparisons. ALT, alanine aminotransferase; AST, aspartate aminotransferase; BMI, body mass index; SCFA, short-chain fatty acid.

Supplementary Table S4. Full phylum- and genus-level relative abundance comparisons in patients with gastric cancer stratified by *Helicobacter pylori* infection status, with Benjamini–Hochberg false discovery rate-adjusted *q*-values

Variables	*H. pylori* negative (n = 25)	*H. pylori* positive (n = 23)	*p*-value	*q* (BH)	*q (CLR)*
**Phylum**					
Bacteroidetes	27.37±17.94	27.02±23.90	0.584	0.643	0.702
Firmicutes	50.36±19.19	56.37±20.25	0.176	0.622	0.702
Proteobacteria	16.12±18.80	11.59±12.58	0.369	0.622	0.702
Actinobacteria	4.11±10.99	3.16±3.80	0.584	0.643	0.828

Variables	*H. pylori* negative (n = 25)	*H. pylori* positive (n = 23)	*p*-value	*q* (BH)	*q (CLR)*
Verrucomicrobia	1.33±5.25	0.83±2.47	0.703	0.703	0.702
Fusobacteria	0.19±0.71	0.66±2.78	0.183	0.622	0.702
Euryarchaeota	0.05±0.14	0.01±0.02	0.264	0.622	0.702
Synergistetes	0.02±0.03	0.02±0.03	0.457	0.629	0.702
Tenericutes	0.01±0.03	0.02±0.03	0.261	0.622	0.702
Lentisphaerae	0.00±0.01	0.01±0.04	0.396	0.622	0.702
F/B ratio	9.90±31.15	6.06±7.91	0.392	0.622	0.702
**Genus**					
Bacteroides	11.38±15.29	7.83 ± 9.64	0.353	0.890	0.928
Prevotella	8.88±12.00	14.89 ± 25.59	0.563	0.890	1.000
Streptococcus	9.01±11.92	14.65 ± 18.05	0.483	0.890	1.000
Lactobacillus	3.61±7.80	3.49 ± 11.43	0.433	0.890	0.928
Lactococcus	0.12±0.36	0.10 ± 0.36	0.398	0.890	1.000
Eubacterium	3.09±4.13	5.96 ± 11.75	0.584	0.890	1.000
Faecalibacterium	1.53±1.51	4.06 ± 4.95	**0.039**	0.564	0.928
Bifidobacterium	2.82±11.01	0.38 ± 0.57	0.342	0.890	0.928
Ruminococcus	2.05 ± 4.48	0.41 ± 0.68	0.223	0.890	0.928
Enterobacter	1.32 ± 4.00	0.88 ± 2.22	0.269	0.890	0.928
Enterococcus	0.56 ± 2.26	0.56 ± 1.80	0.860	0.900	1.000
Fusobacterium	0.19 ± 0.72	0.69 ± 2.91	0.220	0.890	0.928
Veillonella	2.27 ± 3.45	1.63 ± 3.09	0.327	0.890	0.928
Haemophilus	1.57 ± 5.22	0.94 ± 1.67	0.796	0.900	1.000
Paraprevotella	0.34 ± 0.55	0.18 ± 0.23	0.598	0.890	0.928
Catenibacterium	0.29 ± 0.85	0.21 ± 0.43	0.340	0.890	1.000
Salmonella	0.10 ± 0.33	0.16 ± 0.39	0.900	0.900	1.000
Dorea	0.64 ± 1.20	0.76 ± 1.08	0.788	0.900	1.000
Citrobacter	0.57 ± 2.32	0.19 ± 0.31	0.589	0.890	1.000
Gemella	0.05 ± 0.09	0.19 ± 0.32	0.058	0.564	0.928
Megamonas	0.62 ± 1.73	0.41 ± 0.99	0.050	0.564	0.928
Blautia	0.45 ± 0.61	0.42 ± 0.56	0.893	0.900	0.965
Subdoligranulum	0.08 ± 0.18	0.09 ± 0.21	0.715	0.900	1.000
Anaerostipes	0.10 ± 0.30	0.12 ± 0.31	0.554	0.890	1.000
Fusicatenibacter	0.23 ± 0.31	0.34 ± 0.94	0.820	0.900	1.000
Lachnospira	0.03 ± 0.06	0.14 ± 0.60	0.261	0.890	0.928
Roseburia	1.53 ± 1.59	1.43 ± 1.97	0.877	0.900	1.000
Coprococcus	0.26 ± 0.31	0.29 ± 0.39	0.591	0.890	1.000
Oscillibacter	1.20 ± 1.56	1.39 ± 2.75	0.804	0.900	1.000
Butyricimonas	0.56 ± 1.17	0.25 ± 0.45	0.638	0.890	1.000
Odoribacter	0.06 ± 0.08	0.05 ± 0.06	0.758	0.900	1.000
Phascolarctobacterium	0.20 ± 0.39	0.58 ± 1.37	0.194	0.890	0.928
Megasphaera	0.14 ± 0.50	0.10 ± 0.36	0.662	0.890	1.000
Pseudobutyrivibrio	0.03 ± 0.07	0.03 ± 0.05	0.600	0.890	1.000
Akkermansia	1.40 ± 5.46	0.97 ± 2.96	0.649	0.890	1.000
Klebsiella	2.30 ± 4.71	0.73 ± 2.90	**0.029**	0.564	0.928
Atopobium	0.08 ± 0.11	0.48 ± 1.69	0.649	0.890	1.000
P/B ratio	2.98 ± 5.19	16.82 ± 47.40	0.606	0.890	—
SCFA producer abundance	5.89±10.90	6.32±6.29	0.332	0.890	—

Values are presented as mean ± standard deviation relative abundance (%). *p*-values are from Mann-Whitney U tests. *q* (BH) = Benjamini–Hochberg false discovery rate-adjusted q-value, computed separately within the phylum-level and genus-level families of tests. *q* (CLR) = Benjamini–Hochberg-adjusted *p*-value from Mann-Whitney *U* tests on centered log-ratio-transformed abundances, restricted to taxa present in at least 10% of samples; ratios were not included in this analysis. Bold

values indicate statistical significance ($p<0.05$); no comparison remained significant after false discovery rate correction. F/B, *Firmicutes*-to-*Bacteroidetes* ratio; GC, gastric cancer; P/B, *Prevotella*-to-*Bacteroides* ratio.

Supplementary Table S5. Full phylum- and genus-level relative abundance comparisons in patients with gastric cancer stratified by tumor stage, with Benjamini–Hochberg false discovery rate-adjusted *q*-values

Variables	Stage I (n = 43)	≥ Stage II (n = 19)	*p*-value	*q* (BH)	*q (CLR)*
**Phylum**					
Bacteroidetes	27.33±21.09	24.97 ± 14.83	0.885	0.945	0.918
Firmicutes	54.02 ± 19.99	56.06 ± 17.28	0.813	0.945	0.918
Proteobacteria	13.23 ± 17.17	12.04 ± 13.83	0.897	0.945	0.918
Actinobacteria	4.45 ± 9.12	3.23 ± 3.63	0.861	0.945	0.921
Verrucomicrobia	0.36 ± 0.90	2.45 ± 6.37	0.663	0.945	0.918
Fusobacteria	0.14 ± 0.55	0.78 ± 3.06	0.262	0.893	0.906
Euryarchaeota	0.02 ± 0.07	0.11 ± 0.31	0.424	0.932	0.918
Synergistetes	0.02 ± 0.04	0.03 ± 0.03	0.073	0.801	0.906
Tenericutes	0.02 ± 0.03	0.01 ± 0.01	0.325	0.893	0.906
Lentisphaerae	0.01 ± 0.03	0.00 ± 0.00	0.236	0.893	0.906
F/B ratio	8.48 ± 24.23	3.70 ± 3.15	0.945	0.945	—
**Genus**					
Bacteroides	9.22 ± 13.10	9.41 ± 8.11	0.184	0.833	0.928
Prevotella	12.47 ± 19.76	8.76 ± 13.89	0.939	0.945	0.949
Streptococcus	10.47 ± 13.94	10.36 ± 14.83	0.807	0.945	0.928
Lactobacillus	3.90 ± 10.08	4.72 ± 9.84	0.891	0.945	0.928
Lactococcus	0.07 ± 0.27	0.12 ± 0.40	0.785	0.945	0.949
Eubacterium	3.21 ± 4.12	7.66 ± 12.72	0.127	0.833	0.928
Faecalibacterium	2.61 ± 3.49	4.28 ± 4.98	0.096	0.833	0.928
Bifidobacterium	2.11 ± 8.49	0.60 ± 0.95	0.423	0.833	0.928
Ruminococcus	1.38 ± 3.52	0.61 ± 0.69	0.347	0.833	0.928
Enterobacter	0.96 ± 3.22	0.82 ± 1.96	0.288	0.833	0.928
Enterococcus	0.81 ± 3.60	0.70 ± 1.97	0.795	0.945	0.949
Fusobacterium	0.14 ± 0.56	0.82 ± 3.20	0.136	0.833	0.928
Veillonella	1.81 ± 3.39	2.04 ± 3.33	0.487	0.905	0.989
Haemophilus	1.09 ± 4.01	0.87 ± 1.79	0.909	0.945	0.949
Paraprevotella	0.25 ± 0.32	0.29 ± 0.58	0.760	0.945	0.928
Catenibacterium	0.33 ± 0.74	0.12 ± 0.19	0.675	0.945	0.949
Salmonella	0.07 ± 0.26	0.19 ± 0.42	0.840	0.945	0.928
Dorea	0.67 ± 1.13	0.80 ± 1.10	0.427	0.833	0.928
Citrobacter	1.24 ± 7.56	0.78 ± 2.66	0.376	0.833	0.928
Gemella	0.09 ± 0.21	0.11 ± 0.23	0.305	0.833	0.928
Megamonas	0.78 ± 1.81	0.16 ± 0.54	0.945	0.945	0.949
Blautia	0.54 ± 0.79	0.37 ± 0.34	0.873	0.945	0.928
Subdoligranulum	0.08 ± 0.18	0.11 ± 0.23	0.299	0.833	0.928
Anaerostipes	0.12 ± 0.34	0.11 ± 0.18	0.315	0.833	0.928
Fusicatenibacter	0.23 ± 0.41	0.44 ± 1.02	0.189	0.833	0.928
Lachnospira	0.03 ± 0.06	0.17 ± 0.66	0.938	0.945	0.928
Roseburia	1.58 ± 1.81	1.15 ± 0.99	0.766	0.945	0.928
Coprococcus	0.32 ± 0.39	0.32 ± 0.42	0.867	0.945	0.949
Oscillibacter	1.66 ± 3.53	2.62 ± 3.46	0.101	0.833	0.928
Butyricimonas	0.33 ± 0.68	0.54 ± 1.08	0.933	0.945	0.928
Odoribacter	0.05 ± 0.08	0.04 ± 0.06	0.926	0.945	0.949
Phascolarctobacterium	0.34 ± 0.85	0.40 ± 0.99	0.684	0.945	1.000
Megasphaera	0.16 ± 0.47	0.06 ± 0.08	0.337	0.833	0.928

Variables	Stage I (n = 43)	≥ Stage II (n = 19)	*p*-value	*q* (BH)	*q (CLR)*
Pseudobutyrivibrio	0.03 ± 0.06	0.04 ± 0.06	0.745	0.945	0.967
Akkermansia	0.38 ± 0.97	2.68 ± 6.79	0.313	0.833	0.928
Klebsiella	1.66 ± 4.16	0.39 ± 1.01	0.140	0.833	0.928
Atopobium	0.30 ± 1.25	0.13 ± 0.23	0.412	0.833	0.928
P/B ratio	10.86 ± 35.29	2.35 ± 5.56	0.213	0.833	—
SCFA producer abundance	6.40 ± 9.21	6.88 ± 6.03	0.205	0.833	

Values are presented as mean ± standard deviation relative abundance (%). *p*-values are from Mann-Whitney U tests. *q* (BH) = Benjamini–Hochberg false discovery rate-adjusted q-value, computed separately within the phylum-level and genus-level families of tests. *q* (CLR) = Benjamini-Hochberg-adjusted p-value from Mann-Whitney *U* tests on centered log-ratio-transformed abundances, restricted to taxa present in at least 10% of samples; ratios were not included in this analysis. Bold values indicate statistical significance ($p<0.05$); no comparison remained significant after false discovery rate correction. F/B, *Firmicutes*-to-*Bacteroidetes* ratio; GC, gastric cancer; P/B, *Prevotella*-to-*Bacteroides* ratio.

Supplementary Table S6. Full phylum- and genus-level relative abundance comparisons in patients with gastric cancer stratified by primary tumor lesion location, with Benjamini–Hochberg false discovery rate-adjusted *q*-values

Variables	Upper (n = 16)	Middle (n = 18)	Lower (n=25)	*p*-value	*q* (BH)	*q (CLR)*
**Phylum**						
Bacteroidetes	31.00 ± 21.09	25.56 ± 22.58	25.46 ± 16.69	0.659	0.914	0.763
Firmicutes	57.90 ± 19.37	55.92 ± 22.74	51.30 ± 15.78	0.424	0.914	0.763
Proteobacteria	7.25 ± 8.93	12.86 ± 15.58	15.55 ± 19.34	0.466	0.914	0.790
Actinobacteria	2.76 ± 1.96	3.80 ± 5.23	5.34 ± 11.45	0.918	0.918	0.790
Verrucomicrobia	0.69 ± 2.10	0.25 ± 0.62	1.85 ± 5.47	**0.048**	0.530	0.763
Fusobacteria	0.05 ± 0.09	0.78 ± 3.15	0.21 ± 0.71	0.905	0.918	0.944
Euryarchaeota	0.03 ± 0.09	0.02 ± 0.06	0.04 ± 0.13	0.722	0.914	0.763
Synergistetes	0.03 ± 0.03	0.03 ± 0.06	0.02 ± 0.02	0.596	0.914	0.763
Tenericutes	0.02 ± 0.04	0.02 ± 0.04	0.01 ± 0.01	0.376	0.914	0.763
Lentisphaerae	0.00±0.00	0.01 ± 0.05	0.01 ± 0.02	0.748	0.914	0.944
F/B ratio	5.48 ± 8.54	5.20 ± 6.52	9.64 ± 30.96	0.680	0.914	—
**Genus**						
Bacteroides	8.57 ± 15.65	8.98 ± 13.31	10.02±8.28	0.187	0.664	0.855
Prevotella	19.29 ± 20.60	9.86 ± 22.26	8.12 ± 12.58	0.130	0.601	0.613
Streptococcus	13.79 ± 15.34	12.30 ± 16.73	7.92 ± 11.56	0.554	0.924	0.953
Lactobacillus	3.40 ± 8.17	1.69 ± 3.01	5.26 ± 12.02	0.728	0.924	0.953
Lactococcus	0.04 ± 0.11	0.04 ± 0.15	0.09 ± 0.34	0.721	0.924	0.953
Eubacterium	2.99 ± 3.45	6.92 ± 12.57	3.56 ± 5.34	0.320	0.828	0.855
Faecalibacterium	3.92 ± 3.82	2.03 ± 1.70	3.72 ± 5.27	0.318	0.828	0.737
Bifidobacterium	0.56 ± 0.94	0.36 ± 0.35	3.37 ± 11.05	0.340	0.828	0.921
Ruminococcus	0.92 ± 2.34	0.42 ± 0.61	1.43 ± 3.61	0.856	0.954	0.953
Enterobacter	0.21 ± 0.28	1.14 ± 2.78	1.22 ± 3.86	0.982	0.982	0.976
Enterococcus	0.57 ± 2.00	0.24 ± 0.85	1.38 ± 4.68	0.319	0.828	0.953
Fusobacterium	0.05 ± 0.10	0.81 ± 3.29	0.22 ± 0.72	0.706	0.924	0.976
Veillonella	0.99 ± 1.15	1.32 ± 3.01	2.71 ± 4.16	0.139	0.601	0.855
Haemophilus	0.69 ± 1.68	0.41 ± 0.82	1.63 ± 5.21	0.885	0.959	0.976
Paraprevotella	0.34 ± 0.62	0.17 ± 0.17	0.28 ± 0.39	0.804	0.954	0.976
Catenibacterium	0.60 ± 1.01	0.19 ± 0.54	0.12 ± 0.25	0.185	0.664	0.737
Salmonella	0.06 ± 0.17	0.05 ± 0.11	0.12 ± 0.36	0.646	0.924	0.976
Dorea	0.98 ± 0.97	0.73 ± 1.41	0.58 ± 1.03	0.093	0.601	0.520
Citrobacter	0.09 ± 0.19	0.80 ± 2.73	2.09 ± 9.91	0.464	0.838	0.953
Gemella	0.18 ± 0.33	0.10 ± 0.21	0.04 ± 0.10	0.124	0.601	0.737
Megamonas	0.73 ± 1.58	0.31 ± 0.73	0.77 ± 2.02	0.972	0.982	0.976

Variables	Upper (n = 16)	Middle (n = 18)	Lower (n=25)	*p*-value	*q* (BH)	*q (CLR)*
Blautia	0.48 ± 0.60	0.33 ± 0.41	0.66 ± 0.89	0.424	0.838	0.953
Subdoligranulum	0.07 ± 0.18	0.10 ± 0.23	0.10 ± 0.19	0.473	0.838	0.953
Anaerostipes	0.15 ± 0.35	0.12 ± 0.35	0.09 ± 0.23	0.735	0.924	0.976
Fusicatenibacter	0.18 ± 0.19	0.23 ± 0.32	0.42 ± 0.99	0.843	0.954	0.953
Lachnospira	0.21 ± 0.72	0.03 ± 0.06	0.03 ± 0.06	0.470	0.838	0.803
Roseburia	1.35 ± 0.91	1.34 ± 2.27	1.48 ± 1.44	0.412	0.838	0.803
Coprococcus	0.52 ± 0.48	0.26 ± 0.34	0.27 ± 0.37	0.050	0.601	0.466
Oscillibacter	1.09 ± 1.30	2.56 ± 5.34	1.91 ± 2.63	0.818	0.954	0.976
Butyricimonas	0.18 ± 0.44	0.74 ± 1.29	0.30 ± 0.52	0.206	0.669	0.855
Odoribacter	0.05 ± 0.06	0.05 ± 0.06	0.05 ± 0.08	0.680	0.924	0.953
Phascolarctobacterium	0.51 ± 1.10	0.15 ± 0.32	0.43 ± 1.06	0.397	0.838	0.855
Megasphaera	0.18 ± 0.62	0.15 ± 0.42	0.08 ± 0.17	0.725	0.924	0.976
Pseudobutyrivibrio	0.03 ± 0.04	0.03 ± 0.07	0.04 ± 0.07	0.949	0.982	0.976
Akkermansia	0.72 ± 2.23	0.27 ± 0.72	2.02 ± 5.83	**0.044**	0.601	0.466
Klebsiella	0.48 ± 0.99	1.20 ± 3.35	1.39 ± 3.92	0.618	0.924	0.953
Atopobium	0.17 ± 0.38	0.59 ± 1.90	0.07 ± 0.15	0.063	0.601	0.585
P/B ratio	13.70 ± 30.70	13.75 ± 46.64	1.73 ± 4.26	0.061	0.601	—
SCFA producer abundance	6.53 ± 4.41	3.94 ± 3.87	8.97 ± 11.81	0.125	0.601	—

Values are presented as mean ± standard deviation relative abundance (%). *p*-values are from Kruskal-Wallis tests. *q* (BH) = Benjamini–Hochberg false discovery rate-adjusted q-value, computed separately within the phylum-level and genus-level families of tests. *q* (CLR) = Benjamini–Hochberg-adjusted *p*-value from Kruskal-Wallis tests on centered log-ratio-transformed abundances, restricted to taxa present in at least 10% of samples; ratios were not included in this analysis. Bold values indicate statistical significance ($p<0.05$); no comparison remained significant after false discovery rate correction. F/B, *Firmicutes*-to-*Bacteroidetes* ratio; GC, gastric cancer; P/B, *Prevotella*-to-*Bacteroides* ratio.

Figures

(a)

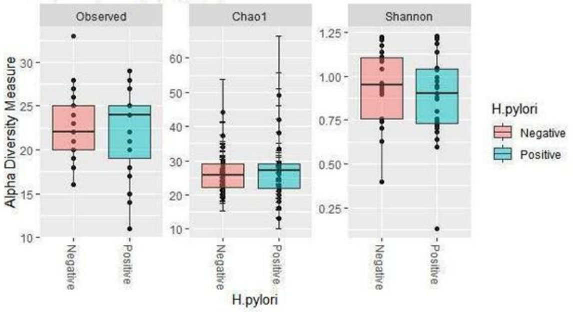

(b)

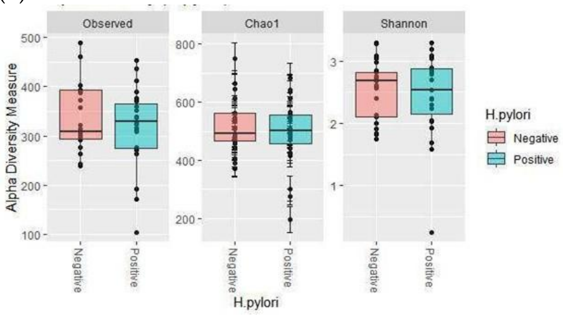

(c)

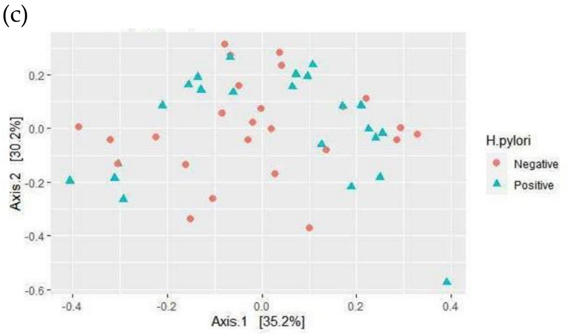

(d)

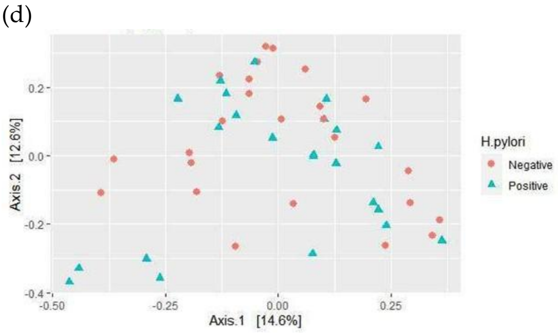

(e)

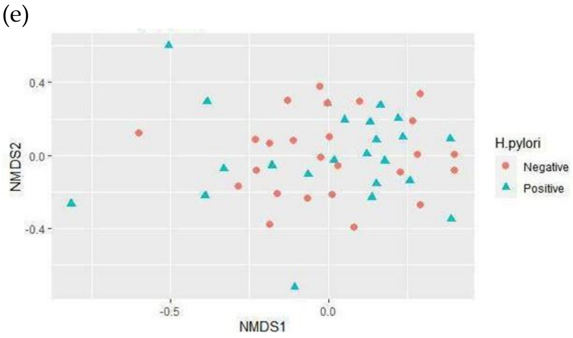

(f)

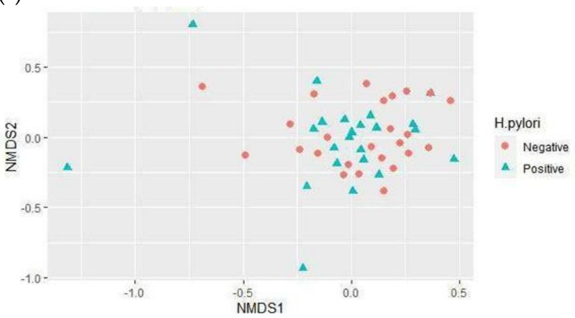

Supplementary Figure S1. Alpha and beta diversity of fecal microbiota in patients with gastric cancer stratified by *Helicobacter pylori* infection status (negative, n = 25; positive, n = 23). Alpha diversity indices (observed OTUs, Chao1 richness, Shannon diversity) at the phylum (a) and genus (b) levels. Beta diversity visualized by Bray–Curtis principal coordinates analysis (PCoA) at the phylum (c) and genus (d) levels. Bray–Curtis non-metric multidimensional scaling (NMDS) ordination at the phylum (e) and genus (f) levels. No alpha diversity index or beta diversity ordination differed significantly between groups.

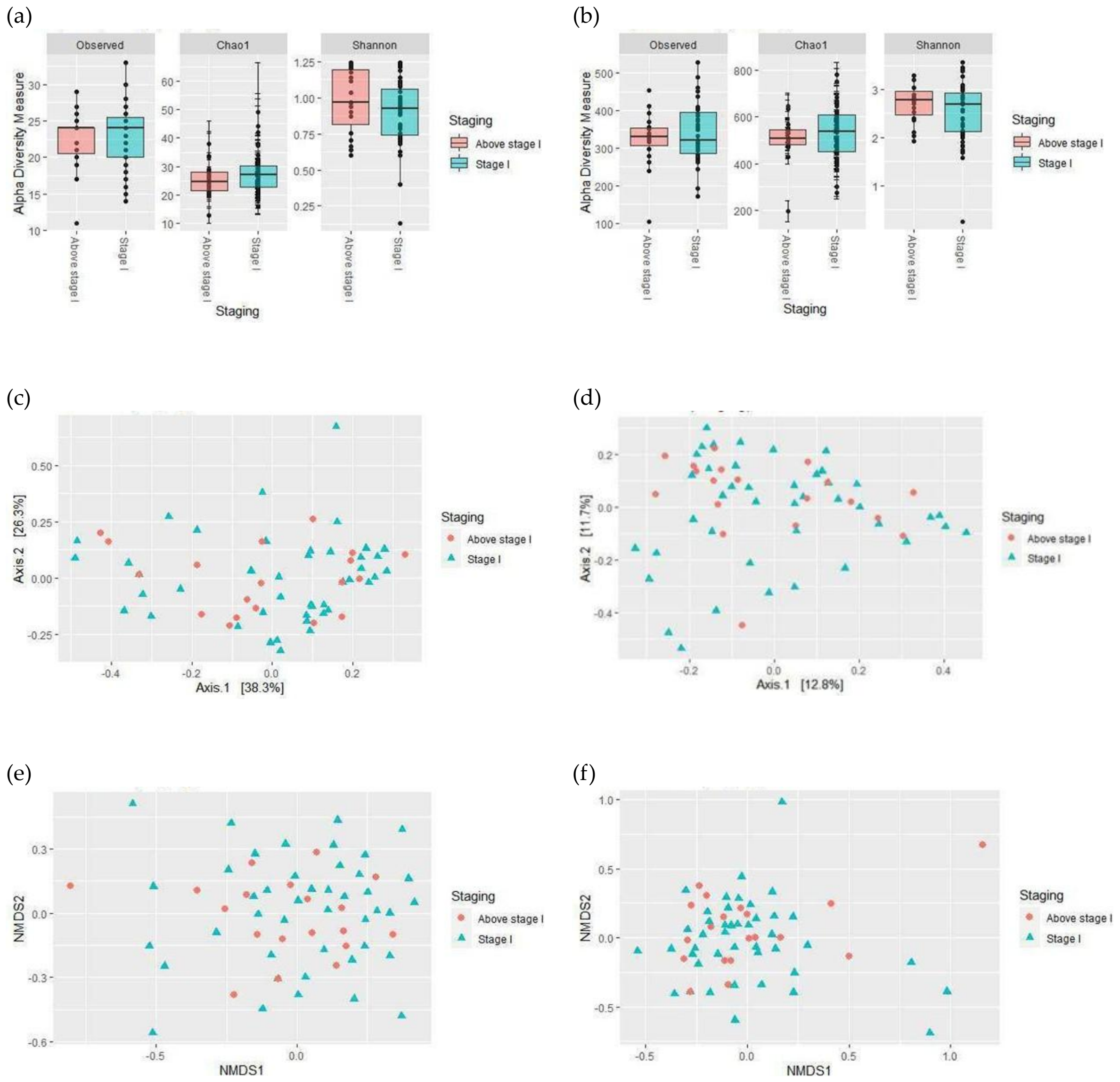

Supplementary Figure S2. Alpha and beta diversity of fecal microbiota in patients with gastric cancer stratified by tumor stage (Stage I, n = 43; ≥ Stage II, n = 19). Alpha diversity indices (observed OTUs, Chao1 richness, Shannon diversity) at the phylum (a) and genus (b) levels. Beta diversity visualized by Bray–Curtis principal coordinates analysis (PCoA) at the phylum (c) and genus (d) levels. Bray–Curtis non-metric multidimensional scaling (NMDS) ordination at the phylum (e) and genus (f) levels. No alpha diversity index or beta diversity ordination differed significantly between groups.

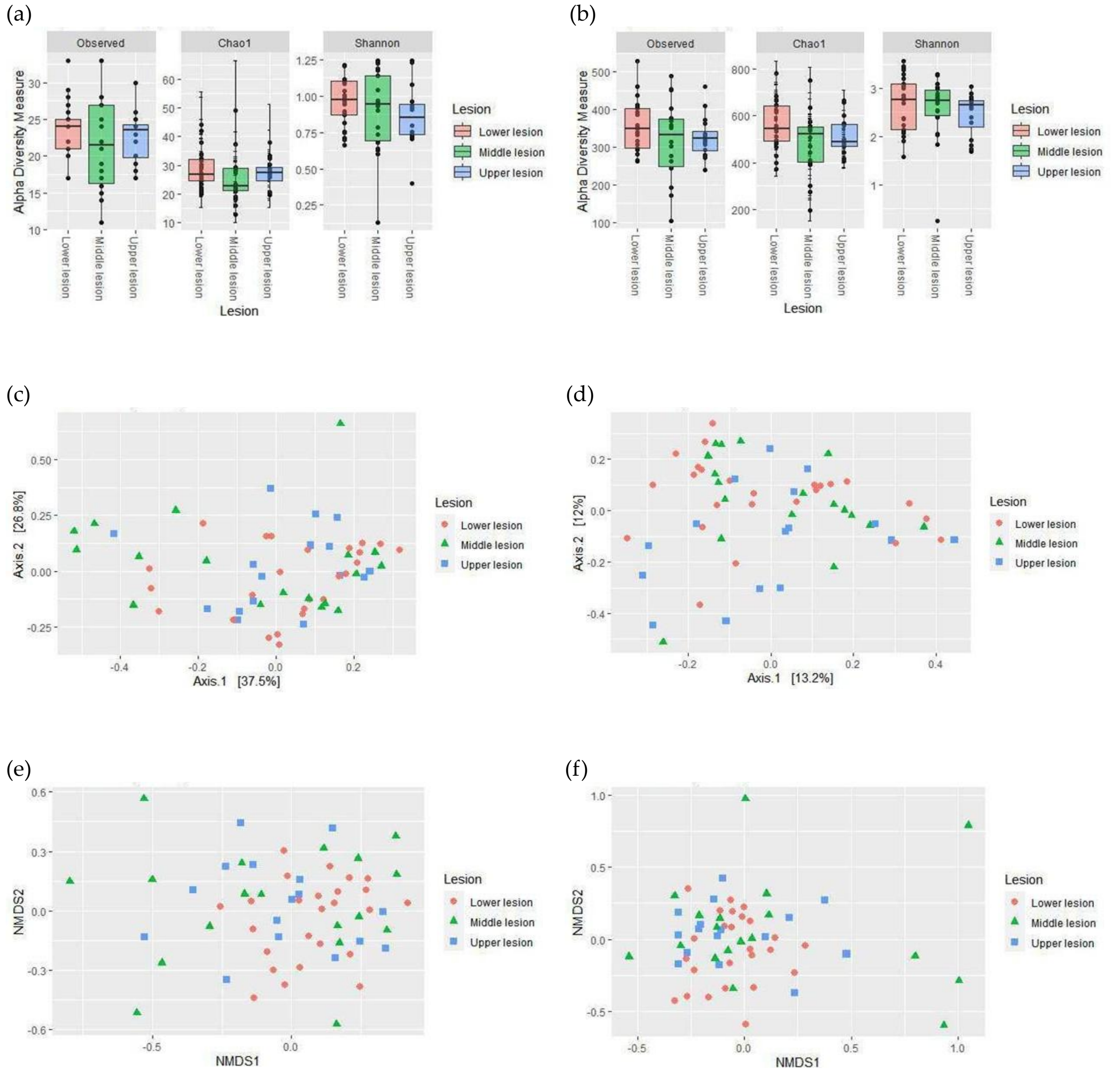

Supplementary Figure S3. Alpha and beta diversity of fecal microbiota in patients with gastric cancer stratified by primary tumor lesion location (upper, n = 16; middle, n = 18; lower, n = 25). Alpha diversity indices (observed OTUs, Chao1 richness, Shannon diversity) at the phylum (a) and genus (b) levels. Beta diversity visualized by Bray–Curtis principal coordinates analysis (PCoA) at the phylum (c) and genus (d) levels. Bray–Curtis non-metric multidimensional scaling (NMDS) ordination at the phylum (e) and genus (f) levels. No alpha diversity index or beta diversity ordination differed significantly across groups.
